# *Trans*-chalcone activity against *Trichophyton rubrum* relies on an interplay between signaling pathways related to cell wall integrity and fatty acid metabolism

**DOI:** 10.1186/s12864-019-5792-0

**Published:** 2019-05-22

**Authors:** Tamires Aparecida Bitencourt, Claudia Macedo, Matheus Eloy Franco, Marina Campos Rocha, Igor Sawasaki Moreli, Bruna Aline Micheloto Cantelli, Pablo Rodrigo Sanches, Rene Oliveira Beleboni, Iran Malavazi, Geraldo Aleixo Passos, Mozart Marins, Ana Lúcia Fachin

**Affiliations:** 1 Unidade de Biotecnologia, Universidade de Ribeirão Preto, Av: Costábile Romano 2201, Ribeirão Preto, SP 14096-900 Brazil; 2 Departamento de Genética, Faculdade de Medicina de Ribeirão Preto, Universidade de São Paulo, Ribeirão Preto, SP Brazil; 3 Departamento de Genética e Evolução, Centro de Ciências Biológicas e da Saúde (CCBS), Universidade Federal de São Carlos, São Carlos, Brazil; 4 Instituto Federal do Sul de Minas - Campus Machado, Machado, Brazil

**Keywords:** Chalcone, CWI, Elastin, Keratin, Dermatophyte, Transcriptional profile

## Abstract

**Background:**

*Trichophyton rubrum* is the main etiological agent of skin and nail infections worldwide. Because of its keratinolytic activity and anthropophilic nature, infection models based on the addition of protein substrates have been employed to assess transcriptional profiles and to elucidate aspects related to host-pathogen interactions. Chalcones are widespread compounds with pronounced activity against dermatophytes. The toxicity of *trans*-chalcone towards *T. rubrum* is not fully understood but seems to rely on diverse cellular targets. Within this context, a better understanding of the mode of action of *trans*-chalcone may help identify new strategies of antifungal therapy and reveal new chemotherapeutic targets. This work aimed to assess the transcriptional profile of *T. rubrum* grown on different protein sources (keratin or elastin) to mimic natural infection sites and exposed to *trans*-chalcone in order to elucidate the mechanisms underlying the antifungal activity of *trans-*chalcone.

**Results:**

Overall, the use of different protein sources caused only slight differences in the transcriptional profile of *T. rubrum*. The main differences were the modulation of proteases and lipases in gene categories when *T. rubrum* was grown on keratin and elastin, respectively. In addition, some genes encoding heat shock proteins were up-regulated during the growth of *T. rubrum* on keratin. The transcriptional profile of *T. rubrum* exposed to *trans*-chalcone included four main categories: fatty acid and lipid metabolism, overall stress response, cell wall integrity pathway, and alternative energy metabolism. Consistently, *T. rubrum* Mapk was strongly activated during the first hours of *trans*-chalcone exposure. Noteworthy, *trans-*chalcone inhibited genes involved in keratin degradation. The results also showed effects of *trans*-chalcone on fatty acid synthesis and metabolic pathways involved in acetyl-CoA supply.

**Conclusion:**

Our results suggest that the mode of action of *trans*-chalcone is related to pronounced changes in fungal metabolism, including an imbalance between fatty acid synthesis and degradation that interferes with cell membrane and cell wall integrity. In addition, this compound exerts activity against important virulence factors. Taken together, *trans*-chalcone acts on targets related to dermatophyte physiology and the infection process.

**Electronic supplementary material:**

The online version of this article (10.1186/s12864-019-5792-0) contains supplementary material, which is available to authorized users.

## Background

Dermatophytes are a group of filamentous fungi that cause cutaneous infections in humans (anthropophilic) and animals (zoophilic). *Trichophyton rubrum* is the most common etiological agent of clinical cases of human dermatophytoses worldwide [1]. The infection generally involves the skin and is restricted to the cornified layers such as nails, stratum corneum, and hair. Although not lethal, dermatophytoses can compromise the quality of life of the affected individual [2].

Because of their keratinolytic and keratinophilic activity, a myriad of endo- and exoproteases have been proposed as the major virulence factors of dermatophytes. Within this context, acid and alkaline proteases are fundamental for nutrient uptake from the insoluble cornified substrates. These proteases are regulated by the simultaneous co-expression of pH signaling genes and regulatory heat shock proteins [3]. Seven dermatophytes genomes have been sequenced [4, 5], which will provide the basis for a better understanding of their pathophysiological mechanisms. Additionally, in vitro and ex vivo models that mimic host-fungal interactions have been employed in order to identify new molecular targets [6].

There is current interest in identifying new molecular targets for antifungal development since most commercially available compounds target the ergosterol biosynthetic pathway and/or cell membrane [7]. In this respect, attention has been drawn to chalcones because of their multiple fungal targets such as enzymes involved in cell wall synthesis concomitant with the inhibition of fatty acid synthesis and reduction of ergosterol content [8]. A co-culture assay of *T. rubrum* conidia with keratinocytes exposed to *trans*-chalcone demonstrated the down-regulation of known virulence factors and genes of the ergosterol pathway [9]. For these reasons, chalcones are attractive molecules with multiple fungal targets. The understanding of the mode of action of chalcones may help identify new strategies of antifungal therapy and these compounds could be used as a pharmacological probe to investigate promising fungal targets.

This study assessed the transcriptional profile of *T. rubrum* during growth on different protein sources (keratin- or elastin) that mimic the host milieu in order to elucidate the mechanisms involved in the activity of *trans-*chalcone against this dermatophyte. Our results indicated that *trans-*chalcone inhibits important virulence factors such as proteases and lipases and causes impairment in essential metabolic pathways and consequent disturbance of cell wall integrity.

## Results

### Data analysis

**Fig. 1:**
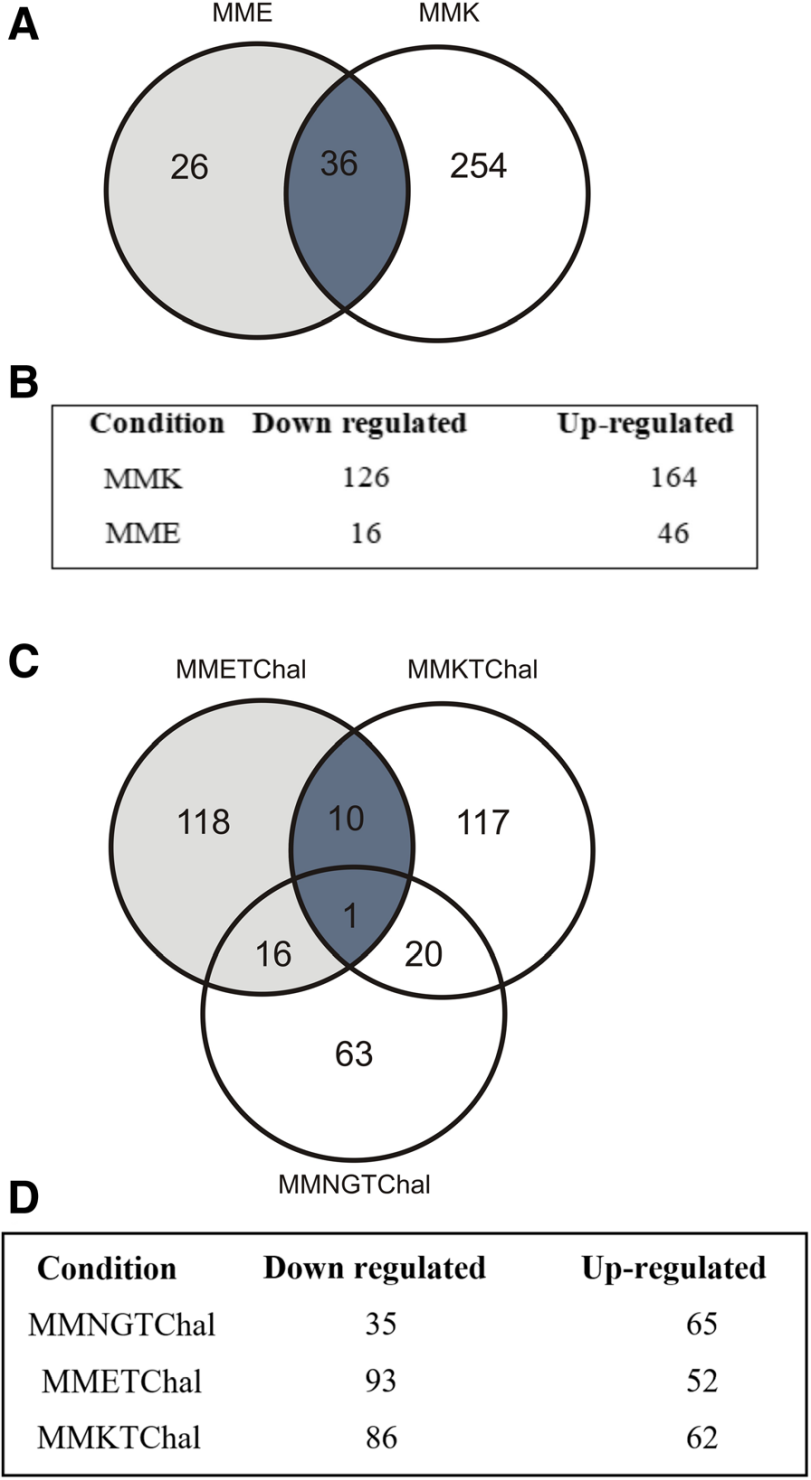
Distribution of gene modulation among the conditions analyzed. (**a**)Venn diagram illustrating the modulation of genes during the growth of *Trichophyton rubrum* on elastin (MME) and keratin (MMK) compared to control (MMNG). (**b**) Box illustration of down- and up-regulated genes comparing the protein sources with MMNG. (**c**) Venn diagram illustrating the modulation of genes after exposure to *trans*-chalcone during growth on protein (MME + TChal and MMK + TChal) or glucose and nitrate (MMNG+TChal) compared to control (same conditions without the drug). (**d**) Box illustration of down- and up-regulated genes comparing the *trans*-chalcone conditions with no drug conditions

The gene expression pattern of *T. rubrum* mycelia grown on protein substrates and exposed to *trans*-chalcone was assessed using high-density oligonucleotide microarray slides that cover about 6091 genes [10], corresponding to about 70% of coding genes in the *T. rubrum* genome (http://fungi.ensembl.org/info/website/ftp/index.html). A total of 290 genes were modulated on keratin medium compared to minimal medium (control) and 62 genes were modulated on elastin medium compared to control. Noteworthy, a fewer genes modulated in elastin condition in comparison to keratin (Fig. 1).

After *trans*-chalcone exposure there were 393 genes modulated, in which 145, 148 and 100 genes modulated on minimal medium supplemented with *trans*-chalcone in the presence of elastin or keratin or minimal medium with glucose and nitrogen, respectively. In general, few genes were shared between conditions. Despite of MMETChal and MMKTChal showed a close number of modulated genes, changes in transcript levels were quite different, in which higher levels were shown for MMKTChal in comparison to MMETChal (Fig. 1, Additional file 1: Table S1).

### Functional categorization of *T. rubrum* genes involved in the interaction with keratin and elastin substrates

**Fig. 2:**
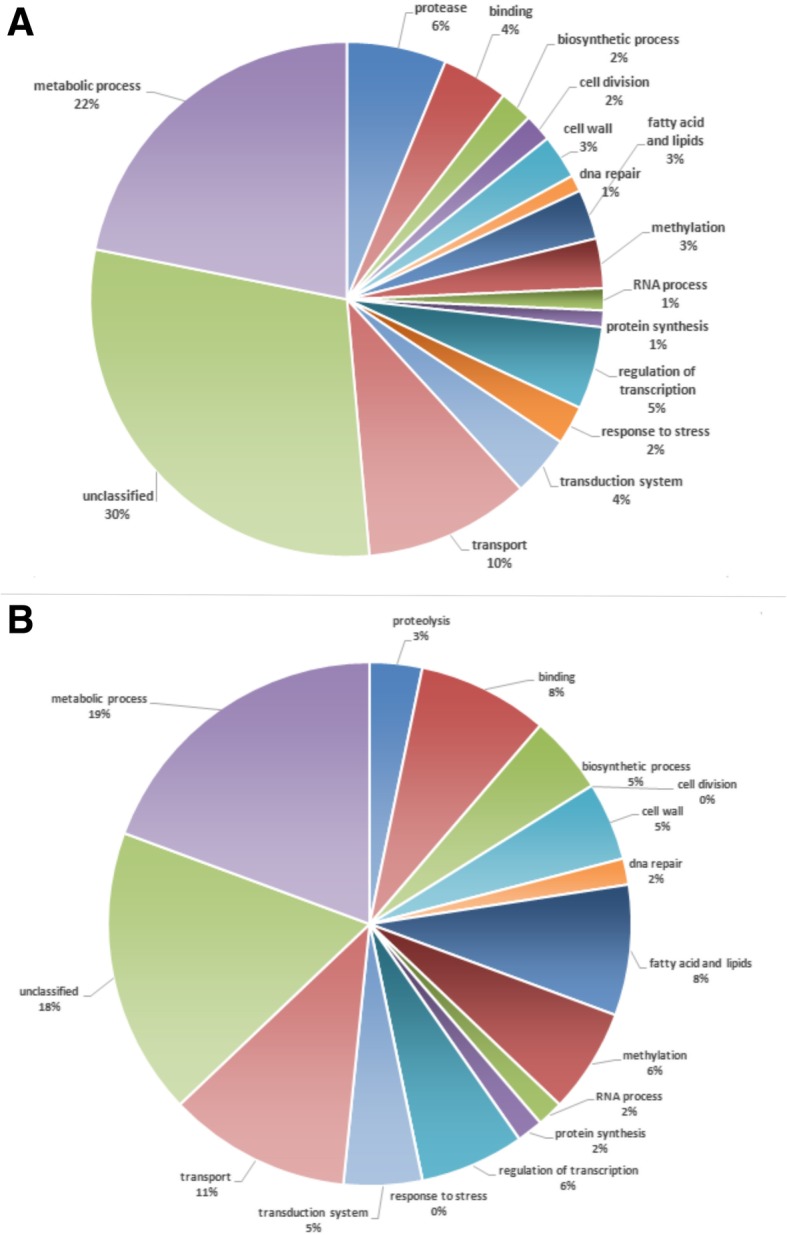
Functional categorization of differentially expressed genes (*p* < 0.05) on MMK (**a**) and MME (**b**)

The functional categorization of differentially expressed genes was performed by gene ontology (GO) using Blast2GO [11]. During *T. rubrum* growth on protein sources, the main categories modulated were related to signal transduction, fatty acid and lipid metabolism, proteolysis, regulation of transcription, transport, metabolic processes, and an elevated number of hypothetical proteins with unknown functions (Fig. 2). Overall, growth on different protein sources caused only slight differences in the gene profile of *T. rubrum*. For instance, *T. rubrum* grown on keratin showed enrichment for genes belonging to the proteolysis and stress response categories. On the other hand, enrichment for genes involved in fatty acid and lipid metabolism, transcription regulation process and cell wall components was observed when elastin was the protein source used (Fig. 2). Accordingly, 18 proteases and 7 lipases were differentially expressed in the two protein source conditions. This finding supports the involvement of protease secretion in keratin utilization in *T. rubrum* (Table 1).

**Table 1:** Protease- and lipase-encoding genes modulated during the growth of *Trichophyton rubrum* on keratin and elastin

ID	Gene product name	Keratin	Elastin
TERG_05923	Metallopeptidase	−6.10	–
TERG_03293	Hypothetical protein	−6.32	–
TERG_04809	Metalloproteinase 2	− 8.12	–
TERG_05652	Leucine aminopeptidase 1	+ 20.71	+ 8.05
TERG_04324	Metalloproteinase 4	+ 24.01	–
TERG_12606	Secreted dipeptidyl peptidase	+ 5.03	–
TERG_03400	Subtilisin-like protease 1	+ 5.17	–
TERG_03104	Signal peptidase i	+ 5.49	–
TERG_06552	Aspartic-type endopeptidase	+ 5.82	–
TERG_04769	Serine carboxypeptidase	+ 5.82	–
TERG_03248	Metalloproteinase 3	+ 6.72	–
TERG_02214	Carboxypeptidase 2	+ 6.79	–
TERG_08557	Carboxypeptidase s1	+ 7.24	–
TERG_08405	Leucine aminopeptidase 2	+ 8.31	–
TERG_05735	Dipeptidyl peptidase 4	+ 8.54	–
TERG_08201	Subtilisin-like protease 5	+ 9.06	–
TERG_03815	Subtilisin-like protease 3	+ 9.88	–
TERG_01617	Subtilisin-like protease 4	+ 9.98	–
TERG_01957	Alkaline serine protease	–	+ 6.52
TERG_03459	GDSL lipase acylhydrolase	–	+ 5.32
TERG_05317	Lipase 1	+ 11.54	–
TERG_04914	Spo7-like protein	+ 5.40	–
TERG_00899	Neutral ceramidase	+ 5.66	–
TERG_00127	Secretory phospholipase a2	+ 52.72	+ 7.01
TERG_03747	Phospholipase a2	+ 6.79	–

+: induction; −: repression

### Functional categorization of *T. rubrum* genes involved in the response to *trans*-chalcone

**Fig. 3:**
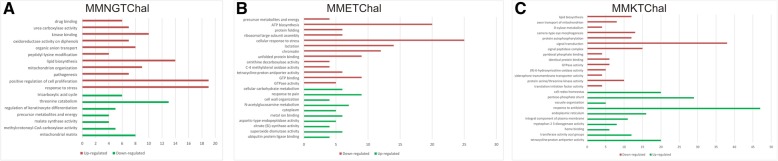
Gene ontology-based functional categorization of representative differentially expressed genes after *trans*-chalcone exposure (p < 0.05) on MMNGTChal (**a**), MMETChal (**b**) and MMKTChal (**c**). The green and red bars indicate up-regulation and down-regulation categories, respectively

The gene expression profile of *T. rubrum* exposed to *trans*-chalcone was assessed to elucidate the mechanisms triggered by this chalcone. Genes above a cut off threshold of 5 and − 5 in fold change in gene expression were submitted to Blast2GO, and then to a summarization using Revigo [12] algorithm. The most representative differentially expressed categories based on GO functions are shown in Fig. 3. Overall, these genes are related to signal transduction, fatty acid and lipid metabolism, response to stress, pathogenesis, cell wall biosynthesis, and metabolic processes.

Noteworthy, *trans*-chalcone exposure down-regulated most genes involved in fatty acid synthesis (Table 2). In addition, genes involved in signal transduction like the cell wall integrity (CWI) pathway and Tor-mediated signaling were also modulated. These finding suggests a cross-talk between pathways to sense and respond to cellular stress caused by *trans*-chalcone (Table 2). Furthermore, alternative routes of energy metabolism were modulated after *trans*-chalcone exposure, such as beta-oxidation of fatty acids and glyoxylate cycle (Table 2).

**Table 2:** Main categories modulated in response to *trans*-chalcone* exposure

ID	Gene product name	Fold change	Condition
*Transduction signaling*			
TERG_01124	RAN-interacting protein	+ 5.37	MMK + TChal
TERG_05617	Hypothetical protein	−5.29	MMK + TChal
TERG_05744	GTP-binding protein	−6.87	MMK + TChal
TERG_02263	Hypothetical protein	−5.29	MMK + TChal
TERG_04042	Serine threonine protein kinase	−8.07	MMK + TChal
TERG_00315	RAN protein kinase	−6.02	MMK + TChal
TERG_00077	Eukaryotic peptide chain release Factor GTP-binding subunit	−6.72	MMK + TChal
TERG_04867	SAM and pH domain-containing protein	−11.78	MMK + TChal
TERG_07136	Farnesyltransferase beta subunit ram1	−9.29/−5.37	MMK + TChal/MMNG+TChal
TERG_05617	Hypothetical protein	−12.39	MMNG+TChal
TERG_00749	Guanine nucleotide exchange	−15.78	MMNG+TChal
TERG_04523	CMGC CDKL CRK7 protein kinase	−5.02	MMNG+TChal
TERG_01365	GTP-binding protein	−5.2	MMNG+TChal
TERG_11963	DEAD/DEAH box RNA helicase	−7.48	MMNG+TChal
TERG_01365	GTP-binding protein	−5.2	MMNG+TChal
TERG_05987	GTP-binding protein	+ 8.17	MMNG+TChal
TERG_01693	Acyl oxidase	−6.42	MMNG+TChal
TERG_02422	RHO GTPase activator	+ 2.18	MME + TChal
TERG_00689	AUR protein kinase	+ 3.34	MME + TChal
TERG_07570	G-protein signaling	−2.35	MME + TChal
TERG_04086	GTP-binding protein 1	−2.36	MME + TChal
TERG_00548	Elongation factor 1 alpha	− 2.6	MME + TChal
TERG_05987	GTP-binding protein	−3.32	MME + TChal
*Fatty acid and lipid metabolism*			
TERG_11538	3-oxoacyl-(acyl-carrier-protein) reductase	−7.45/−10.61	MMK + TChal/MMNG_TChal
TERG_11813	FAD binding domain-containing protein	+ 5.95	MMK + TChal
TERG_11814	FAD dependent protein	+ 10.63	MMK + TChal
TERG_08235	Long-chain fatty alcohol oxidase	−5.52	MMNG+TChal
TERG_04851	Acyl binding protein family	−2.30	MMNG+TChal
TERG_02787	Fatty acid synthase S-acetyl transferase	−5.60	MMNG+TChal
DW707302.1	Enoyl reductase	+ 4.09	MME + TChal
TERG_07644	Ketoacyl reductase	+ 3.38	MME + TChal
*TCA and glyoxylate cycle*			
TERG_03483	Carnitine acetyltransferase	−5.48	MMK + TChal
DW687355.1	Adenylsuccinate lyase	−15.01	MMK + TChal
TERG_05484	Acyl dehydrogenase	+ 6.35	MMK + TChal
TERG_01281	Malate glyoxomal	+ 5.11	MMNG+TChal
TERG_01052	Succinyl ketoacyl transferase	+ 5.1	MMNG+TChal
DW700277.1	Citrate synthase	−6.27	MMNG+TChal
TERG_05484	Acyl dehydrogenase	+ 6.35	MMK + TChal
TERG_04250	Carnitinyl- dehydratase	+ 2.13	MME + TChal
TERG_01271	Isocitrate lyase	+ 2.04	MME + TChal
TERG_01272	Methylcitrate mitochondrial	+ 2.49/+ 5.92	MME + TChal/MMNG+TChal

*TChal: *trans*-chalcone added at 0.24 μg/mL. +: induction; −: repression

### RT-qPCR and Western blot analysis

**Fig. 4:**
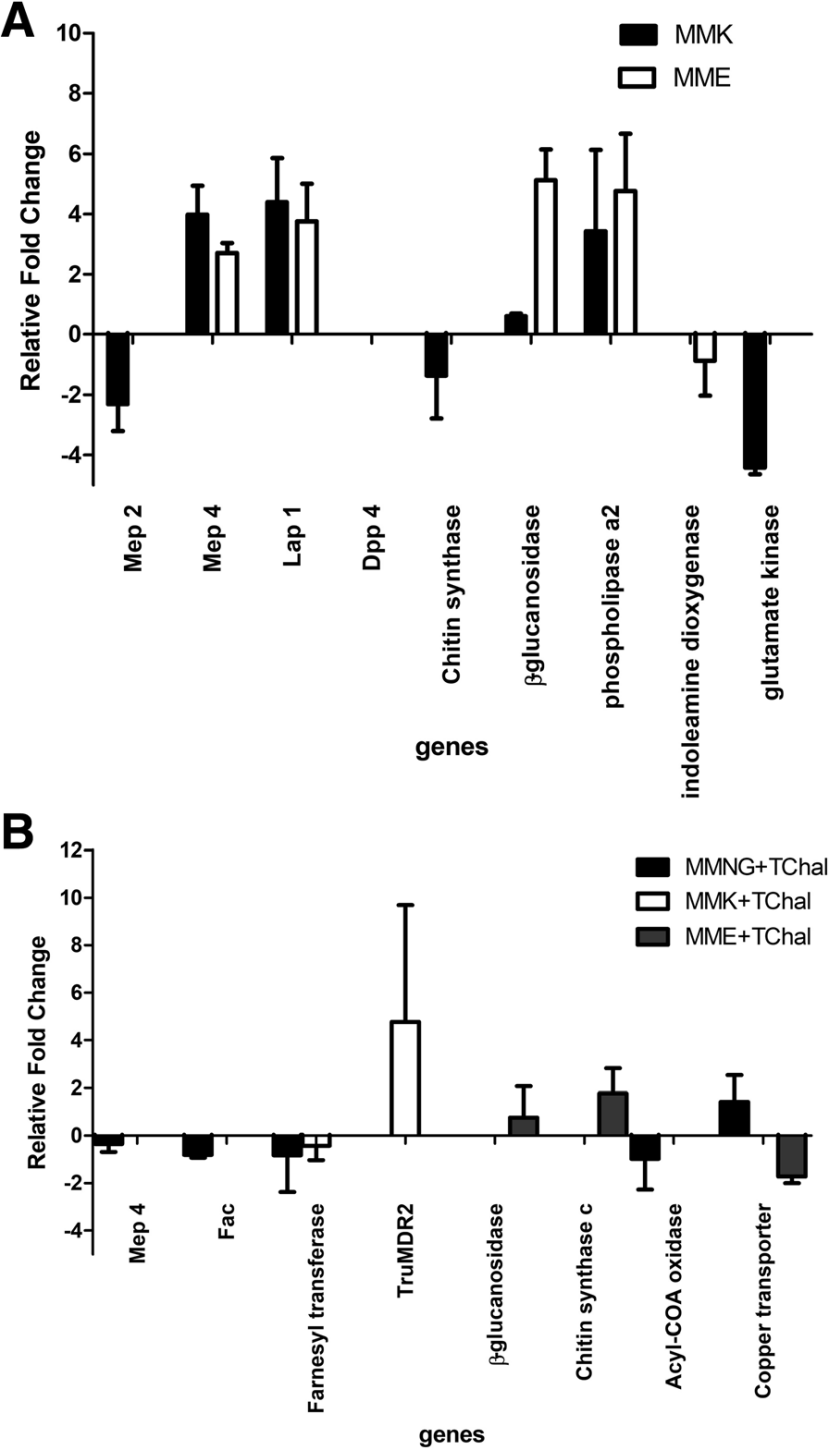
Real-time RT-PCR of selected genes from the microarray hybridization of *Trichophyton rubrum* genes during growth on MMK and MME compared to MMNG (**a**). Modulation of selected genes related to *trans*-chalcone exposure compared to the same condition without drug (**b**). Values are log2 fold change

The microarray results were validated by qPCR and the results demonstrated a strong positive correlation between the two techniques. Pearson correlation r = 0.91 and r = 0.83 for protein sources conditions and trans-chalcone exposure conditions, respectively (Fig. 4). The genes chosen for qPCR analysis were related to different biological processes: (i) proteases (metalloproteases, dipeptidyl protease, leucine aminopeptidase); (ii) lipases (phospholipase); (iii) cell wall biosynthetic pathway (beta glucosidase and chitin synthase); (iv) fatty acid and metabolic processes (fatty acid acetyl transferase, farnesyl transferase, acyl oxidase, copper transporter, glutamate kinase, and indoleamine dioxygenase).

**Fig. 5:**
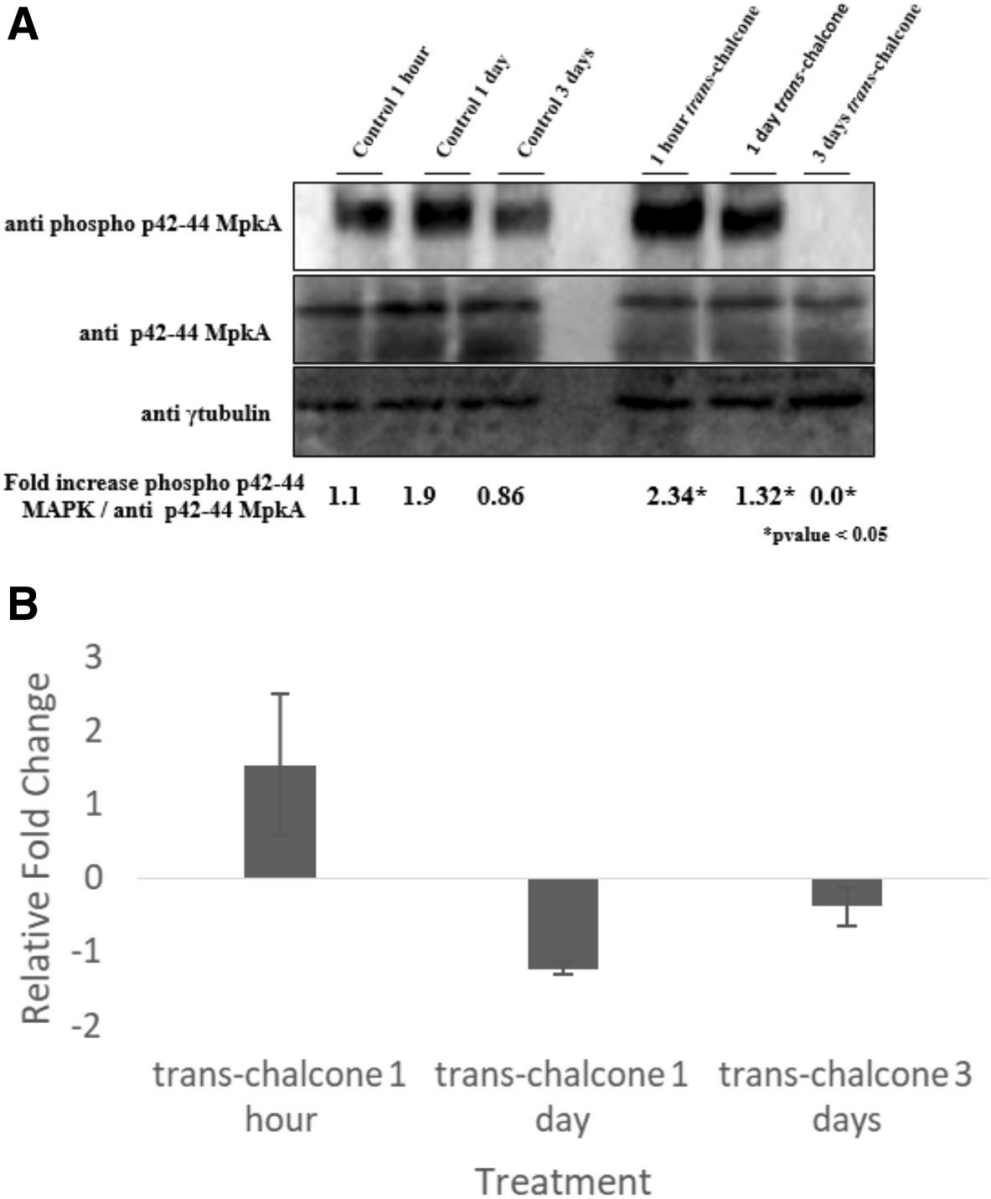
Western blot results of Mapk expression and Mapk phosphorylation. *Trans*-chalcone was added or not (control) to the medium for 1 h, 1 day, and 3 days. Antiphospho-p44/42MAPK antibody was used to detect MpkA phosphorylation. The γ-tubulin antibody was used as control (**a**). Gene expression analysis of CMGC/MAPK Erk1 in *T. rubrum* by qPCR. The fungus was grown in medium with *trans*-chalcone (0.24 μg/mL) or without the compound (control) for 1 h, 1 day, and 3 days (**b**)

In addition to the transcriptional pattern, we also assessed the expression of MAPK after *T. rubrum* exposure to *trans*-chalcone. The gene encoding CMGC MAPK in *T. rubrum* is homologous to MAPK 44/42 in *Aspergillus fumigatus* (score 729, e-value: 0.0, and 85% identity). Here we evaluated the activation of CMGC MAPK by analyzing the phosphorylation levels of this MAP kinase after exposure to *trans*-chalcone. The results showed that MAPK was phosphorylated in response to *trans*-chalcone within the first hour of exposure, followed by a decrease thereafter (1 day). No phosphorylation was observed after 3 days (Fig. 5a). In addition, quantitative RT-PCR was carried out to correlate modulation of the gene encoding CMGC MAPK (TERG_00832) with protein expression (Fig. 5b). This analysis showed up-regulation of *mapk* transcription levels at 1 h, followed by a decrease after 1 day of exposure and little changes in transcription levels after 3 days of exposure to *trans*-chalcone. Noteworthy, our microarray data mainly showed down-regulation of the CWI pathway at the time points analyzed and did not indicate any changes in the modulation of *mapk* gene transcription levels, which probably is due to time points evaluated in our microarray data.

## Discussion

The microarray data suggested slight differences in transcriptional profile of *T. rubrum* grown on different protein sources (keratin or elastin). The main differences were related to the modulation of proteases and some lipases in the keratin condition and to the modulation of genes belonging mainly to the lipase category in the elastin condition. Noteworthy, the low number in genes modulated for elastin substrate may be a result of *T. rubrum* lifestyle, since this fungus is more adapted to use keratin (a component of outermost layer of skin) than elastin (a substrate mainly found in dermis composition). Alike, we believe that the slight changes in transcript levels in MMETChal condition are due to the subtle changes in whole fungal metabolic machinery during *T. rubrum* grown on elastin substrate.

Four main categories can be highlighted in the transcriptional pattern of *T. rubrum* exposed to *trans*-chalcone: fatty acid and lipid metabolism, stress response, CWI pathway, and alternative energy metabolism.

### *Trichophyton rubrum* grown on keratin and elastin focus on proteases, lipases, and heat shock proteins on focus

Simulation of the host milieu is widely employed in investigations to assess putative virulence factors. Within this context, liquid media containing keratin powder, protein and even macerated skin have been used to elucidate key aspects of *T. rubrum* pathophysiology [6].

The establishment of dermatophyte infections is mainly attributed to their ability to adhere to host tissues and to adapt to this environment. Thereafter dermatophytes need to take up nutrients, which is mediated by the induction of specific enzymes such as lipases and proteases, particularly keratinases through sensing of skin pH. Heat shock proteins may be involved in this process through a complex regulatory network in cooperation with PacC transcription factor [13]. Recent reports demonstrated that *pacC*, *hsf1* (heat shock transcription factor), *cdc37* co-chaperone, and *hsp 70* are markedly induced during the growth of dermatophytes on keratin sources at 37 °C [3]. Additionally, in a previous study, the inhibition of Hsp90 decreased the ability of *T. rubrum* to grow on nail fragments [14]. Our microarray data showed that the growth of *T. rubrum* on a keratin source led to the up-regulation of *hsp70-like* (Terg_06505), *hsp90-like* (Terg_06963), and *hsp88-like* (TERG_07658).

Proteases are the most studied virulence factors of dermatophytes as they are required for nutrient uptake during the colonization of host tissue and the abundance of these proteins in the dermatophyte genome highlights their importance in the dermatophyte lifestyle [5, 15]. Proteases are divided into endoproteases and exoproteases. Endoproteases include aspartic proteases, serine proteases (subtilisin family S8A) and metalloproteinases, while the exoprotease group comprises leucine aminopeptidases (Lap 1 and Lap2) and dipeptidyl-peptidases IV and V. Endo- and exoproteases act together in protein digestion [16, 17].

Our data showed that keratin promoted the induction of 15 genes encoding proteases, with expressive induction of the *mep4* and *lap1* genes (Table 1), also demonstrating that exo- and endoproteases are equally important for efficient keratin degradation. Furthermore, exposure to *trans*-chalcone promoted down-regulation of the genes encoding Mep 3, Sub 5, and signal peptidase, as well as of the genes encoding Hsp 70 like-protein, Hsp 88-like protein, and Hsp 90 like-protein (Additional file 1: Table S1).

In the elastin condition, two lipases and two proteases were found to be up-regulated, suggesting that lipases are as important as proteases during *T. rubrum* growth on elastin substrates (Table 1). In this sense, it worth to note that skin composition is also enriched with lipids, and the genomes of *Arthroderma benhaminae* and *T. verrucosum* contain 16 genes encoding lipases [4].

### *Trans*-chalcone exposure shift to the lipid routes by activating alternative energy metabolism

Exposure of *T. rubrum* to *trans*-chalcone promoted changes in lipid and fatty acid metabolism. Genes encoding enzymes involved in the early steps of fatty acid synthesis were generally down-regulated, whereas genes encoding enzymes belonging to the last steps of fatty acid elongation, such as enoyl-reductase and ketoacyl-reductase, were up-regulated (Table 2).

We suggest that impairment of the first steps of fatty acid synthesis led to an imbalance in the pathways involved in the supply of acetyl-CoA molecules. In this respect, β-oxidation is activated in order to supply acetyl-CoA molecules through enzymatic reactions catalyzed by acyl dehydrogenase and ketoacyl-thiolases. The resulting acetyl-CoA can enter the mitochondrial tricarboxylic acid (TCA) cycle [18]. Moreover, citrate from the TCA cycle can be converted to isocitrate, which becomes a glyoxylate cycle substrate, and the resulting malate may enter gluconeogenesis [19]. Consistently, our data showed up-regulation of the genes encoding acyl dehydratases (mediator of first committed steps in fatty acid β oxidation) and ketoacyl thiolases. In addition, exposure to *trans*-chalcone changed the expression of genes related to the glyoxylate and TCA cycles, with up-regulation of the genes encoding isocitrate lyase, succinate lyase, and succinyl-ketoacyl transferase (Table 2).

Moreover a previous work has shown that *trans*-chalcone activity against dermatophytes relies on the down-regulation of fatty acid synthesis β subunit (*fas1*), a decrease in ergosterol content, and moderate inhibition of FAS enzymatic activity [8]. It is noteworthy to mention that palmitate synthesis (the final product of the FAS multienzyme complex [20]) needs the input of acetyl-CoA molecules, reducing agents (NADPH), and energy from ATP. So, its proper synthesis requires the coordinated use of multiple energy metabolic pathways like the TCA and glyoxylate cycles [21]. Supporting this idea, we also demonstrated changes in the modulation of genes involved in acetyl-CoA transport, such as carnitine and citrate synthase.

Taken together, the overall effects promoted by *trans*-chalcone exposure may be related to impairment in fatty acid synthesis and concomitant changes in energy metabolic pathways involved in acetyl-CoA supply. Finally, lipid burning seems to occur as evidenced by changes in the β-oxidation of fatty acids, which confers to *trans*-chalcone a thermogenic feature that might be related to the reduction in ergosterol content and consequent cell wall damage previously described by [8].

### Cross-talk events in the cell wall integrity pathway are activated by *trans*-chalcone

The cell wall is a dynamic structure that is essential to maintain cell shape and to protect against environmental threats. In this regard, the cell wall is remodeled according to developmental stage or after drug exposure. The rearrangements in cell wall composition ensure the structural integrity during conditions that compromise cell wall and/or membrane integrity [22].

The CWI-signaling pathway is activated to protect the cell wall against threats or even during cell growth. The stimuli are sensed by cell wall mechanosensors located on the plasma membrane, such as Mid2 and Mtl1, and by Wsc protein family members. The signals are then transmitted to small Rho1 GTPase, which is activated by regulatory inputs from guanine nucleotide exchange factors (GEFs), Rom1 and Rom2. Next, Rho1p activates protein kinase C (Pkc1). PKC is located upstream in the MAPK (mitogen-activated protein kinase) signaling cascade. Finally, effectors of Rho1 are β-1,3-glucan synthase and β-1,6-glucan synthase, proteins of the actin cytoskeleton and secretory vesicles [22].

During stress, the cell wall undergoes remodeling through a reinforce by increase of chitin amounts and incorporation of certain cell wall proteins. This rearrangement occurs through cross-talk between different signaling pathways [23, 24]. Additionally, any changes in the polarized growth of filamentous fungi are related to impairment of cell wall formation as well as to conditions that indirectly affect cell wall biosynthesis. Thus, to enhance the signaling capabilities of the CWI pathway in order to deal with diverse stress conditions, this pathway cross talks with other signaling pathways or proteins [25]. The interconnection between these responses signaling to pathways of cell wall assembly needs to be widely understood. Within this context, a previous study that compared the response of *A. niger* to caspofungin (inhibitor of β-1,3 glucan synthase) [26] and fenpropimorph (*Saccharomyces cerevisiae* inhibitor of *erg2* and *erg 24* in ergosterol biosynthesis) [27] using the microarray technique showed common responses, promoting changes in signaling pathways such as CWI signaling and Tor signaling and in genes involved in cell membrane composition [24].

In agreement with previous studies, our microarray data showed that exposure of *T. rubrum* to *trans*-chalcone promoted changes in three distinct signaling pathways, suggesting considerable interaction between the Tor signaling, CDK kinase, and MAPK pathways. In addition, Western blot analysis revealed the activation of MAPK after exposure to *trans-*chalcone for 1 h.

## Conclusions

In conclusion, the present results suggest that the mode of action of *trans*-chalcone is related to pronounced changes in fungal metabolism, promoting a shift to lipid metabolism and activating a cross-talk between signaling pathways related to CWI. In addition, we showed that *trans*-chalcone acts on virulence factors such as proteases as well as modulates heat shock proteins. Notwithstanding,we also aware that these interconnections need to be further investigated with association of protein and gene expression profiles.

## Methods

### *Trichophyton rubrum* strain and growth conditions

*Trichophyton rubrum* CBS 118892 was cultured on Sabouraud dextrose agar (Oxoid, Hampshire, England) at 28 °C, as described previously [28]. Conidial suspensions were obtained from 15-day-old plates. The conidial concentration was determined in a Neubauer chamber and approximately 1.6 × 10^6^ conidia were added to 20 mL of liquid Sabouraud and incubated for 72 h at 28 °C under shaking at 150 rpm. The resulting mycelia were incubated under six different conditions: i) control medium (MMNG): Cove’s minimal medium [29] containing 70 mM nitrate (Sigma Aldrich, St. Louis, MO, USA) and 50 mM glucose (Sigma Aldrich); ii) keratin medium (MMK): Cove’s minimal medium supplemented with 0.5% bovine keratin; iii) elastin medium (MME): Cove’s medium supplemented with 0.25% elastin (Sigma Aldrich); iv) MMNG+TChal: MMNG medium containing 0.24 μg/mL of *trans-*chalcone (Sigma Aldrich); v) MMK + TChal: MMK containing 0.24 μg/mL of *trans*-chalcone, and vi) MME + TChal: MME containing 0.24 μg/mL of *trans*-chalcone. The pH of the medium was 5.0 in all conditions and the cultures were incubated for 3, 7, and 14 days at 28 °C under shaking (130 rpm). The concentration of *trans*-chalcone was based on its minimal inhibitory concentration as reported previously [9].

### Total RNA extraction

Total RNA was extracted using the Illustra RNAspin Isolation Kit (GE Healthcare, Little Chalfont, Buckinghamshire, UK) following manufacturer’s instructions. The quality and concentration of the RNA were checked by measuring the OD 260/280 and OD 260/230. RNA degradation was analyzed by microfluidic electrophoresis using Agilent 6000 RNA Nano chips in an Agilent 2100 Bioanalyzer (Agilent Technologies, Santa Clara, CA, USA). Samples with an RNA integrity number (RIN) ≥ 9.0 were used.

### Microarray hybridization

The gene expression profile of *T. rubrum* grown in each of the six conditions was analyzed using the custom slides of the Agilent 4x44K High Density Oligonucleotide Array, as previously described [10]. About thirty-three nanograms of RNA from each incubation time (3, 7 and 14 days) were pooled for each condition. These pooled RNA from each condition was used as a template to generate double strands of cDNA and cRNA labeled with cyanine (Cy3)-CTP using the Agilent Low Input Amplification Kit (Agilent Technologies, Santa Clara, CA, USA). Complementary RNA hybridization was performed using Agilent’s SureHyb chambers in a rotator oven for 18 h at 60 °C. Two biological replicates were used for each condition. In addition to the functional genes of *T. rubrum*, internal control probes were included on the custom slides. The wash steps of the manufacturer’s protocol were followed.

### Analysis of microarray data

The oligo-mRNA array slides were scanned with a DNA microarray scanner (Agilent Technologies) and the Agilent Feature Extraction 10.5 software [30] was used to extract the hybridization signals. The analysis was performed by pairwise comparison using a moderated t-test algorithm as follows: MMK + TChal x MMK; MME + TChal x MME, MMNG+TChal x MMNG, and MMK + TChal x MME + TChal. The quantitative microarray data were normalized by quantile normalization and were analyzed using the Gene Spring GX 12.6 Bioinformatics Platform (http://www.agilent.com/chem/genespring) according to manufacturer’s instructions. Statistical analysis was performed by ANOVA (*P* < 0.05) using a fold change cut-off ≥5.0 for most conditions, except for MME+ TChal in which a fold change ≥2 was used as cut-off. The Benjamini-Hochberg algorithm was used to calculate the false discovery rate, except for comparison between MME + TChal versus MME, in which Storey’s bootstrapping approach was applied*.* The different statistical parameters employed for MME + TChal versus MME is due to the fewer changes in transcript levels for this comparison*.* Each EST with its corresponding protein-coding gene in the *T. rubrum* genome was mapped using Blastx (e-value 1e-5). In addition, putative annotations were retrieved using Blastx and the biological function of mRNA was assessed through GO terms obtained with BayGO [11]. The genes without any associated GO term were called “unclassified”. The raw data are deposited in the Gene Expression Omnibus (GEO) (www.ncbi.nlm.nih.gov/geo) database under accession number: GSE123979.

### RT-qPCR

The microarray expression data were validated by RT-qPCR using a set of 12 genes (Table 3). The oligonucleotide sequences were retrieved from IDT DNA “primer quest” tool (http://www.idtdna.com/primerquest/Home/Index) The selected genes belongs to main categories modulated by *trans*-chalcone exposure or during *T. rubrum* growth on protein sources. Complementary DNA was synthesized from 1000 ng of total RNA in a 20-μL reaction volume using the RevertAID H Minus First Strand cDNA Synthesis Kit (Fermentas®) according to manufacturer’s instruction. The quantitative RT-PCR experiments were performed in triplicate using the SYBR Taq Ready Mix Kit (Sigma) on an Mx3300 QPCR system (Stratagene), as previously described [8]. The cycling conditions included an initial PCR step at 94 °C for 10 min, followed by 40 cycles of 94 °C for 2 min, 60 °C for 1 min, and 72 °C for 1 min. At the end of each PCR cycle, a dissociation curve was constructed. Expression levels were calculated by the comparative 2^-∆∆Ct^ method [31] using beta-tubulin as normalizer. The reference for validation of the microarray data was Cove’s minimal medium for keratin or elastin comparison and the respective condition without *trans*-chalcone for comparison with the drug-containing medium the cultures for 3, 7, and 14 days at 28 °C under shaking (130 rpm). The results are reported as the mean ± standard deviation of three independent experiments.

**Table 3:** Set of primers used in the qPCR assays

ID	Gene product name	Sequence 5′-3’	bp
TERG_11895	Fatty acid acetyl transferase (Fac)	Fwd: 5’-ATGCGCCATGTTCTGTCTCA-3′ Rev.: 5′- TGGTGAAGCGAACAACGAGA-3’	133
TERG_04809	Extracellular metalloproteinase (Mep 2)	Fwd: 5’- GGCACAAGACCAAGAGACCC-3′ Rev.: 5′- AGGCTTGTTGTCCGAGTCAG −3	145
TERG_06242	Beta-glucan glucosidase	Fwd: 5’- CTCAATGTAGCGGCGGGTAT-3′ Rev.: 5′- CACAAAGACTCGGACCCCAA-3’	114
TERG_05652	Aminopeptidase leucine (LAP1)	Fwd: 5’**-** TCCAGGCTGCCATCAATAC-3′ Rev.: 5′- GAATAGTGGCAATGATGCTGTG-3’	99
TERG_02562	Chitin synthase c	Fwd: 5′-TTGCCGGTCTAGGTGTTTAC-3′ Rev.: 5′-CATGCCTATCTGGGTGGTATATT-3’	101
TERG_00694	Glutamate kinase	Fwd: 5’-ATCCTGATGCTCGGGTTATTG-3′ Rev.: 5′-CCACTATCTTTGAGCCCATACC-3’	111
TERG_04324	Extracellular metalloproteinase (Mep4)	Fwd: 5’-GCATGGACTTATGCTTGCGG-3′ Rev.: 5′-TGGATATCTGGGGAAGGCGA	131
TERG_07136	Farnesyl transferase	F:5’-AGGCGTTTACCTTGATCGATAG-3′ R:5′-GCCATCTCCAACTACACCATTA-3’	91
TERG_01329	Cooper transporter	F: 5’- CTCACGGCCAAAGCTATCA-3′ R:5′- TGATCCAGGCGGTGATATTG-3’	105
TERG_02909	Acyl oxidase	F:5’- TGAGAGAGGCCAGTCCAATA-3′ R:5′- TGCTGAATGAGGGAAAGGATAC-3’	102
TERG_00127	Phospholipase a2	F: 5’- GCCACGAGGATACGACTTTAT-3′ R:5′- ATCAACCTTCTTGCGGTAGTC-3’	106
TERG_02134	Indoleamine-dioxygenase	F:5’- CTGCAGCGTATGCCAATAAAG-3′ R:5′- GAGCAGTGAGATCAGGTAACTC-3’	103
TERG_08613	*Tru*MDR2	F: 5’- GCACTGATCTGCAGCTCGACC-3′ R:5’ CCAACGTCATCCTCCCAGAC-3’	91
TERG_00832	CMGC/MAPK protein kinase (Erk1)	F: 5′- CTTGAAGCCCGGTAACCTATT-3’ R: 5′- CGGTCATATATCCAGCGTTCTC −3’	113
TERG_07904	*Beta-tubulin	F: 5’- AACATGATGGCTGCCACTGA-3′ R: 5′ - AAGATGGCAGAGCAGGTAAGGT-3’	253

*Beta tubulin was described by [32]

### Protein extraction and Western blot analysis of phosphorylated MpkA

In order to assess the phosphorylated status of MpkA, a *T. rubrum* conidial suspension was obtained from 15-day-old Sabouraud agar plates. Approximately 1 × 10^7^ conidia were added to 20 mL of liquid Sabouraud under shaken (130 rpm) for 72 h at 28 °C. The mycelia were transferred to minimal medium (MMNG) supplemented with 0.24 μg/mL of *trans*-chalcone and incubated for 1 h, 1 day and 3 days at 28 °C under shaking. The control was left untreated. After incubation, the mycelia were retrieved and frozen at − 80 °C until use. Prior to use, the mycelia were lyophilized. Total protein was extracted and the mycelia were ground in liquid nitrogen with a mortal and a pestle. About 0.5 mL of lysis buffer described in reference [33] was added to the ground mycelium and submitted to vigorous agitation. The lysis buffer contained 10% (v/v) glycerol, 50 mM Tris-HCl, pH 7.5, 1% (v/v) Triton X-100, 150 mM NaCl, 0.1% (w/v) SDS, 5 mM EDTA, 50 mM NaF, 5 mM sodium pyrophosphate, 50 mM β-glycerophosphate, 5 mM sodium orthovanadate, 1 mM PMSF, and 1X Complete Mini Protease Inhibitor (Roche Applied Science). The samples were kept on ice. The extracts were then centrifuged at 20,000 *g* for 1 h at 4 °C. The supernatants were collected and kept at − 80 °C until use. The total protein concentration was determined by the Hartree method [34]. About 50 μg of protein obtained from each condition was resolved on 12% (w/v) SDS-PAGE [35] and transferred to polyvinylidene difluoride (PVDF) membranes (BioRad) using submerged method according to manufacturer’s instructions.

MAP kinase phosphorylation was examined using anti-phospho p44/42 (9101; Cell Signaling Technologies) and anti-p44/42 (9102; Cell Signaling Technologies) antibodies diluted 1:1000 in TBST buffer containing 5% BSA for 16 h at 4 °C, according to manufacturer’s instructions. The primary antibody was detected with HRP-conjugated secondary antibody raised in rabbit (Thermo Scientific) by incubation for 2 h at room temperature. The γ-tubulin antibody was used as the control of the assay. Chemiluminescent detection was conducted using the ECL Prime Western Blot Detection Reagent (GE HealthCare). The images were generated by exposing the membrane to the ChemiDoc XRS gel imaging system (BioRad). The ImageJ software was used for densiometric analysis.

## Additional file

Additional file 1: **Table S1.** Genes modulated by *Trichophyton rubrum* during growth on protein sources, and after trans-chalcone exposure. (DOC 675 kb)

## Abbreviations

CWI: Cell wall Integrity
*fas*: Fatty acid synthase
GEFs: Guanine nucleotide exchange factors
GEO: Gene Expression Omnibus
GO: Gene ontology
MAPK: Mitogen Activated Protein Kinase
MME: Elastin medium
MMETChal: MME containing *trans*-chalcone
MMK: Keratin medium
MMKTChal: MMK containing *trans*-chalcone
MMNG: Cove’s minimal medium containing nitrate and glucose
MMNG+TChal: MMNG medium containing *trans-*chalcone
PKC: protein kinase C
RT-PCR: *Reverse Transcription PCR*
TCA: Tricarboxylic acid
