# Supplementary material for: Trans-chalcone activity against Trichophyton rubrum relies on an interplay between signaling pathways related to cell wall integrity and fatty acid metabolism
Source: BMC Genomics. 2019 May 22;20:411. doi: 10.1186/s12864-019-5792-0 (PMC6532161; doi:10.1186/s12864-019-5792-0)
Supplement: Supplementary file 1 — Table S1. Genes modulated by Trichophyton rubrum during growth on protein sources, and after trans-chalcone exposure. (DOC 675 kb) [file 12864_2019_5792_MOESM1_ESM.doc]

**Additional file 1: Table S1** Genes modulated by *Trichophyton rubrum* during growth on protein sources, and after trans-chalcone exposure

| **ID** | **Puttative annotation** | **Description** | **Geometric mean fold-change (log2)** |
| --- | --- | --- | --- |
| TERG_00041 | hypothetical protein H100_07463 | ElastinXE_Transchalcone | -1.3125 |
| TERG_00072 | hypothetical protein TERG_00072 | ControlXKeratin | 3.0724 |
| TERG_00073 | nadph dehydrogenase | ElastinXE_Transchalcone | -1.1744 |
| TERG_00077 | eukaryotic peptide chain release factor gtp-binding subunit | KeratinXK_Transchalcone | -2.7480 |
| TERG_00114 | protein fam92a1 | ControlXKeratin | 2.4559 |
| TERG_00124 | allergen asp f3 | ElastinXE_Transchalcone | -1.3129 |
| TERG_00127 | secretory phospholipase a2 | ControlXElastin | 2.8102 |
| TERG_00127 | secretory phospholipase a2 | ControlXKeratin | 5.7203 |
| TERG_00127 | secretory phospholipase a2 | KeratinXK_Transchalcone | -2.8813 |
| TERG_00155 | eukaryotic translation initiation factor 1a | KeratinXK_Transchalcone | -2.8781 |
| TERG_00162 | mfs multidrug transporter | ControlXC_Transchalcone | -2.3881 |
| TERG_00162 | mfs multidrug transporter | ControlXKeratin | -2.9143 |
| TERG_00162 | mfs multidrug transporter | KeratinXK_Transchalcone | 2.0364 |
| TERG_00204 | mannitol-1-phosphate 5-dehydrogenase | ControlXKeratin | -2.5823 |
| TERG_00215 | mfs peptide transporter | ControlXElastin | 2.3971 |
| TERG_00216 | endochitinase 1 | ControlXElastin | 2.6840 |
| TERG_00221 | hypothetical protein TRV_03379 | ElastinXE_Transchalcone | -1.0316 |
| TERG_00243 | pathogenesis associated protein | ControlXKeratin | -2.4296 |
| TERG_00243 | pathogenesis associated protein | ElastinXE_Transchalcone | -1.0552 |
| TERG_00243 | pathogenesis associated protein | KeratinXK_Transchalcone | 2.3436 |
| TERG_00271 | hypothetical protein TERG_00271 | ElastinXE_Transchalcone | 1.1415 |
| TERG_00281 | agc rsk protein kinase | ControlXKeratin | 2.4570 |
| TERG_00299 | acetolactate large biosynthetic type | ControlXElastin | 2.9778 |
| TERG_00299 | acetolactate large biosynthetic type | ControlXKeratin | 2.7181 |
| TERG_00315 | ran protein kinase | KeratinXK_Transchalcone | -2.5901 |
| TERG_00334 | hypothetical protein TERG_00334 | KeratinXK_Transchalcone | 2.5732 |
| TERG_00344 | hypothetical protein TERG_00344 | KeratinXK_Transchalcone | 2.5790 |
| TERG_00355 | pyruvate decarboxylase | ElastinXE_Transchalcone | -1.2222 |
| TERG_00355 | pyruvate decarboxylase | KeratinXK_Transchalcone | 2.6497 |
| TERG_00359 | bzip transcription factor | KeratinXK_Transchalcone | 2.3545 |
| TERG_00385 | amid-like nadh | ControlXKeratin | -2.4681 |
| TERG_00456 | cysteine dioxygenase | ElastinXE_Transchalcone | -1.1187 |
| TERG_00487 | hypothetical protein TERG_00487 | ControlXKeratin | 2.8373 |
| TERG_00491 | hypothetical protein TERG_00491 | ElastinXE_Transchalcone | -1.7375 |
| TERG_00520 | hypothetical protein TERG_00520 | ControlXElastin | 2.9456 |
| TERG_00520 | hypothetical protein TERG_00520 | ControlXKeratin | 3.6134 |
| TERG_00523 | hypothetical protein TERG_00523 | ControlXKeratin | -2.6169 |
| TERG_00524 | proline-rich protein -15 | ElastinXE_Transchalcone | -1.1713 |
| TERG_00548 | elongation factor 1-alpha | ElastinXE_Transchalcone | -1.3788 |
| TERG_00553 | amino acid permease | ControlXElastin | 3.2322 |
| TERG_00559 | eukaryotic translation initiation factor 2 alpha subunit | ControlXKeratin | 2.3611 |
| TERG_00563 | gnat family n-acetyltransferase | KeratinXK_Transchalcone | 2.6082 |
| TERG_00566 | hypothetical protein TRV_02989 | ControlXC_Transchalcone | -2.7150 |
| TERG_00566 | hypothetical protein TRV_02989 | ControlXKeratin | -2.5752 |
| TERG_00566 | hypothetical protein TRV_02989 | ElastinXE_Transchalcone | -1.5152 |
| TERG_00583 | extracellular serine-rich protein | ControlXKeratin | 2.5190 |
| TERG_00585 | indoleamine -dioxygenase family protein | KeratinXK_Transchalcone | 2.6842 |
| TERG_00598 | hypothetical protein TERG_00598 | ControlXKeratin | -4.0717 |
| TERG_00668 | l-galactose dehydrogenase (l- ) | ControlXKeratin | -2.3369 |
| TERG_00668 | l-galactose dehydrogenase (l- ) | KeratinXK_Transchalcone | 2.4940 |
| TERG_00689 | aur protein kinase | ElastinXE_Transchalcone | 1.7388 |
| TERG_00694 | glutamate 5-kinase | ControlXKeratin | -3.1070 |
| TERG_00725 | hypothetical protein TERG_00725 | ElastinXE_Transchalcone | 1.4786 |
| TERG_00733 | pre-mrna-splicing factor 38b | ElastinXE_Transchalcone | -1.2608 |
| TERG_00736 | alpha beta hydrolase fold protein | ControlXElastin | 2.5238 |
| TERG_00736 | alpha beta hydrolase fold protein | ControlXKeratin | 2.5887 |
| TERG_00739 | ubiquitin-like protein 1 | ControlXC_Transchalcone | 2.8018 |
| TERG_00749 | guanine nucleotide exchange | ControlXC_Transchalcone | -3.9797 |
| TERG_00749 | guanine nucleotide exchange | ControlXKeratin | -2.3975 |
| TERG_00754 | hypothetical protein H100_08207 | ControlXKeratin | 2.3877 |
| TERG_00776 | mfs transporter | ControlXC_Transchalcone | -3.5274 |
| TERG_00819 | pe repeat family protein | ControlXC_Transchalcone | 3.5416 |
| TERG_00819 | pe repeat family protein | ControlXKeratin | 3.7185 |
| TERG_00852 | phytanoyl- dioxygenase | ControlXC_Transchalcone | -2.9826 |
| TERG_00867 | duf1212 domain membrane protein | ControlXKeratin | -5.0187 |
| TERG_00877 | cbs domain-containing protein | ControlXKeratin | 2.4394 |
| TERG_00880 | actin filament organization protein app1- | ElastinXE_Transchalcone | -1.6257 |
| TERG_00881 | reticulon-4-interacting protein 1 | ElastinXE_Transchalcone | -1.3074 |
| TERG_00887 | hypothetical protein TEQG_00993 | ControlXKeratin | -2.4705 |
| TERG_00899 | neutral ceramidase | ControlXKeratin | 2.5016 |
| TERG_00911 | hypothetical protein TERG_00911 | ControlXC_Transchalcone | -3.1251 |
| TERG_00911 | hypothetical protein TERG_00911 | ControlXElastin | -3.2717 |
| TERG_00911 | hypothetical protein TERG_00911 | ControlXKeratin | 2.3582 |
| TERG_00911 | hypothetical protein TERG_00911 | KeratinXK_Transchalcone | -3.4065 |
| TERG_00942 | calcofluor white hypersensitive protein | KeratinXK_Transchalcone | 2.3423 |
| TERG_00956 | antigenic thaumatin domain | ElastinXE_Transchalcone | -1.2425 |
| TERG_00959 | hypothetical protein TERG_00959 | KeratinXK_Transchalcone | 2.5632 |
| TERG_00967 | hypothetical protein H100_08426 | ControlXKeratin | 3.0816 |
| TERG_00967 | hypothetical protein H100_08426 | KeratinXK_Transchalcone | -2.7406 |
| TERG_00978 | rna processing protein | ControlXKeratin | -2.7420 |
| TERG_01045 | threonine-trna ligase | ControlXC_Transchalcone | -2.5950 |
| TERG_01052 | succinyl- :3-ketoacid-coenzyme a transferase | ControlXC_Transchalcone | 2.3505 |
| TERG_01062 | beta-ig-h3 fasciclin | ElastinXE_Transchalcone | 1.0189 |
| TERG_01104 | hypothetical protein TERG_01104 | ControlXKeratin | 2.5607 |
| TERG_01122 | chaperone heat shock protein | KeratinXK_Transchalcone | 2.9960 |
| TERG_01124 | ran-interacting protein | ControlXKeratin | -2.7593 |
| TERG_01124 | ran-interacting protein | KeratinXK_Transchalcone | 2.4256 |
| TERG_01126 | beta- -glucanosyltransferase 3 | ControlXKeratin | 3.1213 |
| TERG_01126 | beta- -glucanosyltransferase 3 | KeratinXK_Transchalcone | -2.6207 |
| TERG_01150 | 2-heptaprenyl- -naphthoquinone | ControlXElastin | -3.3793 |
| TERG_01150 | 2-heptaprenyl- -naphthoquinone | ControlXKeratin | -2.9806 |
| TERG_01151 | proteasome regulatory particle subunit | ControlXC_Transchalcone | -2.3745 |
| TERG_01154 | parasitic phase-specific protein psp-1 | ControlXC_Transchalcone | -2.3418 |
| TERG_01190 | rna binding protein | KeratinXK_Transchalcone | 2.6578 |
| TERG_01252 | catalase a | ControlXKeratin | -2.9832 |
| TERG_01271 | isocitrate lyase | ElastinXE_Transchalcone | 1.0324 |
| TERG_01272 | 2-methylcitrate mitochondrial | ControlXC_Transchalcone | 2.5660 |
| TERG_01272 | 2-methylcitrate mitochondrial | ControlXKeratin | 4.1284 |
| TERG_01272 | 2-methylcitrate mitochondrial | ElastinXE_Transchalcone | 1.3156 |
| TERG_01276 | choline transporter | KeratinXK_Transchalcone | 2.4500 |
| TERG_01281 | malate glyoxysomal | ControlXC_Transchalcone | 2.3536 |
| TERG_01281 | malate glyoxysomal | ControlXKeratin | -2.5587 |
| TERG_01329 | copper transporter | ControlXC_Transchalcone | 2.7447 |
| TERG_01329 | copper transporter | ElastinXE_Transchalcone | -1.7722 |
| TERG_01330 | expression library immunization antigen 1 | ElastinXE_Transchalcone | -1.4790 |
| TERG_01339 | glutamate decarboxylase | ControlXC_Transchalcone | 3.0894 |
| TERG_01339 | glutamate decarboxylase | ControlXElastin | 3.3443 |
| TERG_01339 | glutamate decarboxylase | ControlXKeratin | -2.8076 |
| TERG_01339 | glutamate decarboxylase | ElastinXE_Transchalcone | -2.2804 |
| TERG_01353 | v-type c subunit family protein | ControlXKeratin | 2.3903 |
| TERG_01364 | hypothetical protein TESG_07982 | ControlXKeratin | -4.2472 |
| TERG_01365 | gtp-binding protein | ControlXC_Transchalcone | -2.3791 |
| TERG_01365 | gtp-binding protein | ControlXElastin | -2.8194 |
| TERG_01365 | gtp-binding protein | ControlXKeratin | -5.2229 |
| TERG_01365 | gtp-binding protein | ElastinXE_Transchalcone | 1.3923 |
| TERG_01475 | dna topoisomerase 2 | ControlXKeratin | 2.5214 |
| TERG_01480 | multidrug transporter | ControlXKeratin | 2.5944 |
| TERG_01480 | multidrug transporter | KeratinXK_Transchalcone | -2.7866 |
| TERG_01494 | hypothetical protein H105_08958 | ControlXKeratin | -2.8106 |
| TERG_01503 | duf895 domain membrane protein | ControlXElastin | 2.9080 |
| TERG_01503 | duf895 domain membrane protein | ControlXKeratin | 2.9677 |
| TERG_01503 | duf895 domain membrane protein | ElastinXE_Transchalcone | 1.3166 |
| TERG_01505 | swim zinc finger domain protein | ControlXKeratin | -2.4668 |
| TERG_01523 | hypothetical protein H100_09001 | KeratinXK_Transchalcone | 2.8156 |
| TERG_01529 | phosphoglycerate mutase | ControlXKeratin | 2.5462 |
| TERG_01591 | small nucleolar ribonucleoprotein complex subunit | ControlXElastin | 3.6687 |
| TERG_01599 | hypothetical protein TERG_01599 | ControlXC_Transchalcone | 1.7600 |
| TERG_01599 | hypothetical protein TERG_01599 | ControlXElastin | 5.8781 |
| TERG_01599 | hypothetical protein TERG_01599 | ControlXKeratin | 7.0352 |
| TERG_01599 | hypothetical protein TERG_01599 | ElastinXE_Transchalcone | 1.0997 |
| TERG_01600 | cytochrome c oxidase-assembly factor mitochondrial | ControlXKeratin | 3.4357 |
| TERG_01604 | 60s ribosomal protein l36 | ElastinXE_Transchalcone | -1.1038 |
| TERG_01609 | na k atpase alpha 1 | ControlXKeratin | -2.9176 |
| TERG_01617 | subtilisin-like protease 4 | ControlXKeratin | 3.3187 |
| TERG_01617 | subtilisin-like protease 4 | ElastinXE_Transchalcone | 1.0959 |
| TERG_01623 | major facilitator superfamily transporter | KeratinXK_Transchalcone | 2.7309 |
| TERG_01641 | fk506 suppressor sfk1 | KeratinXK_Transchalcone | 2.6857 |
| TERG_01645 | mitochondrial carrier protein | KeratinXK_Transchalcone | 2.4857 |
| TERG_01657 | family a g protein-coupled receptor-like protein | ControlXC_Transchalcone | -3.5697 |
| TERG_01657 | family a g protein-coupled receptor-like protein | ControlXKeratin | -2.4924 |
| TERG_01659 | 30 kda heat shock protein | ControlXC_Transchalcone | -3.1785 |
| TERG_01659 | 30 kda heat shock protein | ControlXKeratin | 2.9203 |
| TERG_01668 | duf396 doamin protein | ControlXKeratin | -2.5952 |
| TERG_01731 | hypothetical protein TERG_01731 | ControlXKeratin | 3.0646 |
| TERG_01731 | hypothetical protein TERG_01731 | KeratinXK_Transchalcone | -2.4900 |
| TERG_01741 | benzoate 4-monooxygenase cytochrome p450 | KeratinXK_Transchalcone | 2.9508 |
| TERG_01748 | mfs transporter | ControlXElastin | 5.0278 |
| TERG_01748 | mfs transporter | ControlXKeratin | 3.3700 |
| TERG_01749 | hypothetical protein TERG_01749 | ControlXKeratin | 3.5528 |
| TERG_01838 | lim domain-containing protein | ControlXElastin | 3.1789 |
| TERG_01838 | lim domain-containing protein | ElastinXE_Transchalcone | -2.5837 |
| TERG_01914 | cytochrome p450 | ControlXElastin | 2.6042 |
| TERG_01914 | cytochrome p450 | ControlXKeratin | 2.7069 |
| TERG_01925 | 5 3 -nucleotidase | ControlXC_Transchalcone | -2.9304 |
| TERG_01925 | 5 3 -nucleotidase | ControlXElastin | -2.9864 |
| TERG_01925 | 5 3 -nucleotidase | ControlXKeratin | -3.8247 |
| TERG_01957 | alkaline serine protease | ControlXC_Transchalcone | 3.3824 |
| TERG_01957 | alkaline serine protease | ControlXElastin | 2.7049 |
| TERG_01957 | alkaline serine protease | ElastinXE_Transchalcone | -1.3837 |
| TERG_01959 | rna polymerase rpb1 c-terminal repeat domain-containing protein | ControlXKeratin | 2.4079 |
| TERG_01959 | rna polymerase rpb1 c-terminal repeat domain-containing protein | KeratinXK_Transchalcone | -3.1272 |
| TERG_01979 | pirin | ControlXKeratin | 2.7807 |
| TERG_01981 | hypothetical protein TERG_01981 | ControlXC_Transchalcone | -2.7072 |
| TERG_02008 | hypothetical protein ARB_06520 | ControlXElastin | 3.1932 |
| TERG_02008 | hypothetical protein ARB_06520 | ElastinXE_Transchalcone | 1.3341 |
| TERG_02039 | 2-hydroxyphytanoyl- lyase | ControlXC_Transchalcone | 2.4635 |
| TERG_02079 | hypothetical protein H100_06753 | ControlXKeratin | -2.5087 |
| TERG_02079 | hypothetical protein H100_06753 | ElastinXE_Transchalcone | -1.2034 |
| TERG_02107 | protein phosphatase type 1 complex subunit hex2 | ControlXKeratin | -2.6178 |
| TERG_02112 | hypothetical protein TERG_02112 | KeratinXK_Transchalcone | -2.4958 |
| TERG_02118 | gpi anchored | ControlXKeratin | 2.5547 |
| TERG_02132 | 5-histidylcysteine sulfoxide synthase | ControlXC_Transchalcone | -2.6594 |
| TERG_02132 | 5-histidylcysteine sulfoxide synthase | KeratinXK_Transchalcone | -3.1505 |
| TERG_02133 | flug protein | KeratinXK_Transchalcone | -2.5612 |
| TERG_02134 | indoleamine -dioxygenase-like protein | ControlXC_Transchalcone | -2.4258 |
| TERG_02134 | indoleamine -dioxygenase-like protein | ControlXElastin | -2.3297 |
| TERG_02134 | indoleamine -dioxygenase-like protein | KeratinXK_Transchalcone | -2.7976 |
| TERG_02139 | eukaryotic translation initiation factor 3 subunit d | ControlXKeratin | 3.1236 |
| TERG_02177 | snp3 protein | ElastinXE_Transchalcone | 1.7805 |
| TERG_02214 | carboxypeptidase 2 | ControlXKeratin | 2.7629 |
| TERG_02230 | mitochondrial ribosomal protein dap3 | ControlXElastin | 2.8397 |
| TERG_02237 | 40s ribosomal protein s28 | ControlXElastin | 3.3982 |
| TERG_02263 | hypothetical protein TERG_02263 | KeratinXK_Transchalcone | -2.4055 |
| TERG_02278 | hypothetical protein ARB_02458 | ControlXElastin | -4.9102 |
| TERG_02278 | hypothetical protein ARB_02458 | KeratinXK_Transchalcone | -2.5511 |
| TERG_02280 | hexokinase | ControlXKeratin | -2.8301 |
| TERG_02333 | acetyl-coenzyme a transporter 1 | KeratinXK_Transchalcone | 2.7735 |
| TERG_02367 | hypothetical protein H105_07045 | ControlXKeratin | 2.9881 |
| TERG_02367 | hypothetical protein H105_07045 | KeratinXK_Transchalcone | -2.6167 |
| TERG_02406 | alpha-box mating type protein | ControlXElastin | 2.4153 |
| TERG_02406 | alpha-box mating type protein | ControlXKeratin | 3.4258 |
| TERG_02412 | hhe domain protein | ControlXC_Transchalcone | -3.1217 |
| TERG_02412 | hhe domain protein | ControlXKeratin | -5.5289 |
| TERG_02422 | rho gtpase activator | ElastinXE_Transchalcone | 1.1250 |
| TERG_02449 | phosphoadenosine phosphosulfate reductase | ElastinXE_Transchalcone | 1.3399 |
| TERG_02493 | glyoxalase family protein | ControlXKeratin | 2.3902 |
| TERG_02496 | histone h1 | ControlXKeratin | 2.7403 |
| TERG_02500 | phosphatidylinositol-4-phosphate 5-kinase its3 | ControlXC_Transchalcone | -3.5256 |
| TERG_02500 | phosphatidylinositol-4-phosphate 5-kinase its3 | ControlXElastin | -2.3755 |
| TERG_02528 | ubie coq5 methyltransferase | ControlXKeratin | -2.5831 |
| TERG_02528 | ubie coq5 methyltransferase | KeratinXK_Transchalcone | 2.9756 |
| TERG_02541 | fad binding domain-containing protein | ControlXC_Transchalcone | 2.4214 |
| TERG_02545 | mfs monocarboxylate transporter | ControlXKeratin | -2.3873 |
| TERG_02562 | chitin synthase | ElastinXE_Transchalcone | 1.1000 |
| TERG_02603 | cue domain | ControlXC_Transchalcone | 2.9324 |
| TERG_02603 | cue domain | ElastinXE_Transchalcone | -1.6401 |
| TERG_02659 | non-classical export protein nce102 | ControlXKeratin | 2.5525 |
| TERG_02659 | non-classical export protein nce102 | ElastinXE_Transchalcone | 1.0784 |
| TERG_02678 | protein-l-isoaspartate o-methyltransferase | ControlXKeratin | -2.4549 |
| TERG_02684 | nudix domain protein | KeratinXK_Transchalcone | 2.6538 |
| TERG_02711 | nonribosomal peptide synthase pes1 | ControlXKeratin | -2.4872 |
| TERG_02712 | glutamyl-trna amidotransferase | ControlXC_Transchalcone | -2.6598 |
| TERG_02719 | glycosyl hydrolase | ControlXKeratin | 3.0795 |
| TERG_02722 | wsc domain containing protein | ControlXKeratin | 3.0957 |
| TERG_02722 | wsc domain containing protein | KeratinXK_Transchalcone | -2.5524 |
| TERG_02783 | mfs monocarboxylate | ControlXC_Transchalcone | -2.6589 |
| TERG_02785 | hypothetical protein TERG_02785 | ControlXC_Transchalcone | -2.3896 |
| TERG_02791 | sphingolipid long chain base-responsive protein pil1 | ControlXKeratin | -2.6331 |
| TERG_02794 | hypothetical protein TERG_02794 | KeratinXK_Transchalcone | 2.7899 |
| TERG_02809 | hypothetical protein TERG_02809 | ControlXElastin | 2.8439 |
| TERG_02809 | hypothetical protein TERG_02809 | ControlXKeratin | -2.7581 |
| TERG_02820 | polyketide synthase | ControlXKeratin | -3.8671 |
| TERG_02841 | aflatoxin cluster transcription partial | ControlXC_Transchalcone | -3.0853 |
| TERG_02841 | aflatoxin cluster transcription partial | ControlXKeratin | -3.6826 |
| TERG_02842 | 6-hydroxy-d-nicotine oxidase | ControlXC_Transchalcone | -2.5988 |
| TERG_02842 | 6-hydroxy-d-nicotine oxidase | ControlXKeratin | -4.8940 |
| TERG_02842 | 6-hydroxy-d-nicotine oxidase | KeratinXK_Transchalcone | -2.7437 |
| TERG_02843 | fungal specific transcription factor domain-containing protein | ControlXC_Transchalcone | -3.1828 |
| TERG_02843 | fungal specific transcription factor domain-containing protein | ControlXKeratin | -3.6787 |
| TERG_02843 | fungal specific transcription factor domain-containing protein | ElastinXE_Transchalcone | -1.3691 |
| TERG_02844 | major facilitator superfamily transporter | ControlXKeratin | -4.3329 |
| TERG_02844 | major facilitator superfamily transporter | ElastinXE_Transchalcone | -1.0494 |
| TERG_02844 | major facilitator superfamily transporter | KeratinXK_Transchalcone | -2.4011 |
| TERG_02846 | multicopper oxidase | ControlXC_Transchalcone | -3.0509 |
| TERG_02846 | multicopper oxidase | ControlXKeratin | -2.8995 |
| TERG_02848 | short chain dehydrogenase reductase family protein | ControlXC_Transchalcone | -2.7837 |
| TERG_02848 | short chain dehydrogenase reductase family protein | ControlXKeratin | -2.5971 |
| TERG_02848 | short chain dehydrogenase reductase family protein | KeratinXK_Transchalcone | -2.4103 |
| TERG_02886 | hypothetical protein TERG_02886 | ControlXKeratin | -2.8082 |
| TERG_02902 | hypothetical protein TERG_02902 | KeratinXK_Transchalcone | 2.3368 |
| TERG_02953 | hypothetical protein H100_05221 | ControlXC_Transchalcone | -2.9179 |
| TERG_02958 | assimilatory sulfite reductase | ControlXKeratin | -2.3447 |
| TERG_02960 | low quality protein: phenolpthiocerol synthesis polyketide synthase ppsb | KeratinXK_Transchalcone | 2.5822 |
| TERG_02974 | hypothetical protein TERG_02974 | ControlXKeratin | -2.7758 |
| TERG_03004 | 3-methyl-2-oxobutanoate hydroxymethyltransferase | ControlXKeratin | 2.5200 |
| TERG_03033 | btb domain and ankyrin repeat protein | ControlXKeratin | -2.7268 |
| TERG_03058 | zinc-binding oxidoreductase | ControlXKeratin | -2.4045 |
| TERG_03059 | protein toxd | ControlXKeratin | -2.4783 |
| TERG_03080 | isoleucyl-trna synthetase | ControlXC_Transchalcone | 2.4360 |
| TERG_03095 | methyltransferase | ControlXElastin | 2.8589 |
| TERG_03095 | methyltransferase | ControlXKeratin | 2.5739 |
| TERG_03104 | signal peptidase i | ControlXKeratin | 2.4558 |
| TERG_03104 | signal peptidase i | ElastinXE_Transchalcone | -1.5543 |
| TERG_03104 | signal peptidase i | KeratinXK_Transchalcone | -2.7077 |
| TERG_03105 | hypothetical protein TERG_03105 | ControlXKeratin | 2.4322 |
| TERG_03105 | hypothetical protein TERG_03105 | ElastinXE_Transchalcone | -1.1522 |
| TERG_03124 | histone h4 | KeratinXK_Transchalcone | -2.6324 |
| TERG_03130 | c2h2 transcription factor | ElastinXE_Transchalcone | -1.0471 |
| TERG_03161 | hypothetical protein H113_05473 | ControlXC_Transchalcone | -3.4705 |
| TERG_03161 | hypothetical protein H113_05473 | ControlXElastin | -2.3757 |
| TERG_03161 | hypothetical protein H113_05473 | ControlXKeratin | 2.3259 |
| TERG_03161 | hypothetical protein H113_05473 | KeratinXK_Transchalcone | -2.9124 |
| TERG_03164 | hypothetical protein H100_05442 | ControlXC_Transchalcone | -2.3623 |
| TERG_03174 | siderochrome-iron transporter sit1 | ControlXKeratin | 2.4595 |
| TERG_03175 | duf833 domain-containing protein | ElastinXE_Transchalcone | -1.2658 |
| TERG_03193 | hypothetical protein H100_05470 | ControlXKeratin | -2.7345 |
| TERG_03211 | actin cytoskeleton protein | ElastinXE_Transchalcone | -1.9233 |
| TERG_03223 | n-acetylglucosamine-6-phosphate deacetylase | ElastinXE_Transchalcone | 1.3760 |
| TERG_03226 | glucosamine-6-phosphate deaminase | ControlXC_Transchalcone | 2.8318 |
| TERG_03226 | glucosamine-6-phosphate deaminase | ControlXKeratin | 2.7300 |
| TERG_03226 | glucosamine-6-phosphate deaminase | ElastinXE_Transchalcone | 1.3674 |
| TERG_03226 | glucosamine-6-phosphate deaminase | KeratinXK_Transchalcone | 3.0361 |
| TERG_03229 | hexokinase | ElastinXE_Transchalcone | 1.0960 |
| TERG_03240 | drug resistance transporter | ElastinXE_Transchalcone | -1.6243 |
| TERG_03248 | extracellular metalloproteinase 3 | ControlXKeratin | 2.7490 |
| TERG_03248 | extracellular metalloproteinase 3 | KeratinXK_Transchalcone | -2.3647 |
| TERG_03252 | hypothetical protein H105_05540 | ControlXKeratin | 3.7678 |
| TERG_03252 | hypothetical protein H105_05540 | ElastinXE_Transchalcone | 1.5650 |
| TERG_03274 | ammonium transporter | ControlXKeratin | -2.9313 |
| TERG_03293 | hypothetical protein TERG_03293 | ControlXKeratin | -2.6598 |
| TERG_03304 | aaa family | ElastinXE_Transchalcone | 1.0091 |
| TERG_03314 | taurine catabolism dioxygenase | ControlXC_Transchalcone | -2.7240 |
| TERG_03314 | taurine catabolism dioxygenase | ControlXKeratin | -2.3367 |
| TERG_03327 | er membrane protein | KeratinXK_Transchalcone | 2.5913 |
| TERG_03339 | alternative oxidase | ElastinXE_Transchalcone | -1.2133 |
| TERG_03340 | short chain dehydrogenase reductase family protein | ControlXKeratin | -2.5063 |
| TERG_03400 | subtilisin-like protease 1 | ControlXKeratin | 2.3711 |
| TERG_03452 | heat repeat protein | ControlXKeratin | -2.3392 |
| TERG_03457 | hypothetical protein TERG_03457 | ControlXKeratin | 5.2246 |
| TERG_03459 | gdsl lipase acylhydrolase family protein | ControlXElastin | 2.4124 |
| TERG_03459 | gdsl lipase acylhydrolase family protein | ControlXKeratin | 3.1069 |
| TERG_03459 | gdsl lipase acylhydrolase family protein | ElastinXE_Transchalcone | 1.2437 |
| TERG_03475 | diphthamide biosynthesis protein 1 | ControlXElastin | -3.4480 |
| TERG_03475 | diphthamide biosynthesis protein 1 | ControlXKeratin | -2.4043 |
| TERG_03483 | carnitine acetyl transferase | KeratinXK_Transchalcone | -2.4535 |
| TERG_03492 | oxalate decarboxylase | ElastinXE_Transchalcone | 1.3100 |
| TERG_03506 | hypothetical protein TERG_03506 | ElastinXE_Transchalcone | -1.3065 |
| TERG_03553 | v-type proton atpase subunit b | ControlXKeratin | 3.3279 |
| TERG_03556 | transcriptional regulator medusa | ControlXKeratin | 2.5662 |
| TERG_03599 | metalloproteinase | ElastinXE_Transchalcone | -1.2263 |
| TERG_03602 | polysaccharide deacetylase family protein | ControlXKeratin | -2.3889 |
| TERG_03621 | hypothetical protein H100_05929 | ControlXKeratin | 4.5245 |
| TERG_03625 | hypothetical protein H101_07251 | ControlXKeratin | 2.5525 |
| TERG_03662 | hypothetical protein TERG_03662 | KeratinXK_Transchalcone | -2.7202 |
| TERG_03676 | ring finger domain-containing protein | ControlXKeratin | -2.3255 |
| TERG_03676 | ring finger domain-containing protein | ElastinXE_Transchalcone | -1.7921 |
| TERG_03677 | protein phosphatase regulatory subunit | ControlXKeratin | -2.3287 |
| TERG_03702 | hypothetical protein TERG_03702 | ElastinXE_Transchalcone | 1.0519 |
| TERG_03705 | cytochrome p450 | ControlXKeratin | 2.9333 |
| TERG_03729 | 6-phosphogluconate dehydrogenase | KeratinXK_Transchalcone | 3.1321 |
| TERG_03730 | rta1 domain | KeratinXK_Transchalcone | 3.8380 |
| TERG_03738 | 60s ribosomal protein l3 | ControlXKeratin | 2.8873 |
| TERG_03747 | phospholipase a2 | ControlXKeratin | 2.7645 |
| TERG_03761 | hypothetical protein TERG_03761 | ControlXKeratin | -2.5817 |
| TERG_03780 | pyruvate dehydrogenase dihydrolipoamide acetyltransferase component | ControlXKeratin | 3.9671 |
| TERG_03793 | elongation factor 3 | ElastinXE_Transchalcone | -1.0437 |
| TERG_03811 | actin-binding protein | ControlXKeratin | 2.4842 |
| TERG_03815 | subtilisin-like protease 3 | ControlXKeratin | 3.3052 |
| TERG_03815 | subtilisin-like protease 3 | KeratinXK_Transchalcone | -2.3677 |
| TERG_03826 | duf4243 and methyltransferase domain protein | ControlXKeratin | -2.9493 |
| TERG_03837 | pyridoxal-phosphate dependent | ControlXC_Transchalcone | 2.6118 |
| TERG_03840 | bromodomain associated domain protein | ControlXC_Transchalcone | -3.2035 |
| TERG_03847 | s-adenosyl-l-methionine-dependent methyltransferase | ControlXKeratin | -2.6499 |
| TERG_03875 | glycogen phosphorylase | KeratinXK_Transchalcone | -2.6087 |
| TERG_03892 | hypothetical protein TERG_03892 | ControlXKeratin | -3.0567 |
| TERG_03899 | methyltransferase type 11 | ControlXElastin | -2.4365 |
| TERG_03899 | methyltransferase type 11 | ControlXKeratin | -2.9524 |
| TERG_03904 | aflatoxin b1 aldehyde reductase member 2 | ControlXKeratin | -2.4193 |
| TERG_03907 | amino acid transporter | ControlXKeratin | -2.9317 |
| TERG_03934 | ankyrin repeat and zinc finger domain-containing protein 1 | ElastinXE_Transchalcone | -1.0914 |
| TERG_03974 | glycine-rich protein | ControlXKeratin | 2.9277 |
| TERG_03985 | efflux pump antibiotic resistance | ControlXKeratin | 2.9027 |
| TERG_04002 | 60s ribosome biogenesis protein sqt1 | ElastinXE_Transchalcone | -1.1319 |
| TERG_04009 | hypothetical protein TERG_04009 | ControlXC_Transchalcone | -3.2863 |
| TERG_04032 | dna repair and transcription factor ada | ControlXKeratin | 3.5508 |
| TERG_04042 | serine threonine protein kinase | KeratinXK_Transchalcone | -3.0137 |
| TERG_04044 | vacuolar protein sorting protein | ControlXElastin | 3.0443 |
| TERG_04044 | vacuolar protein sorting protein | ControlXKeratin | 2.3377 |
| TERG_04074 | hypothetical protein TERG_04074 | ElastinXE_Transchalcone | -1.1429 |
| TERG_04086 | gtp-binding protein 1 | ElastinXE_Transchalcone | -1.2419 |
| TERG_04093 | k+ homeostasis protein kha1 | KeratinXK_Transchalcone | -2.5192 |
| TERG_04098 | dna repair protein swi5 sae3 | ControlXKeratin | -2.4457 |
| TERG_04130 | plasma membrane atpase | ElastinXE_Transchalcone | -1.2062 |
| TERG_04154 | hypothetical protein TRV_07950 | ControlXKeratin | 2.3807 |
| TERG_04154 | hypothetical protein TRV_07950 | KeratinXK_Transchalcone | -2.6052 |
| TERG_04159 | protein | KeratinXK_Transchalcone | -2.7153 |
| TERG_04179 | hypothetical protein TERG_04179 | ControlXKeratin | 2.3688 |
| TERG_04183 | yqci protein | ControlXKeratin | -3.2292 |
| TERG_04228 | hypothetical protein TERG_04228 | ControlXKeratin | -2.7834 |
| TERG_04234 | hypothetical protein H100_04471 | ControlXC_Transchalcone | 2.3409 |
| TERG_04234 | hypothetical protein H100_04471 | ControlXKeratin | 3.3013 |
| TERG_04234 | hypothetical protein H100_04471 | KeratinXK_Transchalcone | -2.3528 |
| TERG_04240 | cupin domain-containing protein | ControlXC_Transchalcone | -2.4716 |
| TERG_04240 | cupin domain-containing protein | KeratinXK_Transchalcone | -3.0731 |
| TERG_04250 | carnitinyl- dehydratase | ElastinXE_Transchalcone | 1.0894 |
| TERG_04269 | hypothetical protein H105_04517 | ControlXKeratin | 2.7387 |
| TERG_04281 | woronin body major protein | KeratinXK_Transchalcone | -2.4410 |
| TERG_04292 | hypothetical protein TERG_04292 | KeratinXK_Transchalcone | 2.5982 |
| TERG_04294 | methylcrotonoyl- carboxylase subunit beta | ControlXC_Transchalcone | 2.9345 |
| TERG_04308 | mfs sugar transporter | ControlXElastin | -2.6324 |
| TERG_04310 | alcohol dehydrogenase | ControlXKeratin | -2.6436 |
| TERG_04324 | extracellular metalloproteinase 4 | ControlXKeratin | 4.5854 |
| TERG_04335 | superoxide dismutase | ControlXC_Transchalcone | 4.0368 |
| TERG_04335 | superoxide dismutase | ControlXKeratin | 2.4802 |
| TERG_04335 | superoxide dismutase | ElastinXE_Transchalcone | 1.2751 |
| TERG_04400 | monosaccharide transporter | ControlXKeratin | 2.9127 |
| TERG_04402 | glyceraldehyde-3-phosphate dehydrogenase | ControlXKeratin | 2.9644 |
| TERG_04449 | nucleolus protein | ControlXKeratin | -2.5942 |
| TERG_04469 | d-3-phosphoglycerate dehydrogenase | ControlXKeratin | -2.4997 |
| TERG_04487 | hypothetical protein H100_04732 | ControlXKeratin | -2.6491 |
| TERG_04487 | hypothetical protein H100_04732 | KeratinXK_Transchalcone | -2.3412 |
| TERG_04504 | extracellular protein | ElastinXE_Transchalcone | -1.2075 |
| TERG_04523 | cmgc cdk crk7 protein kinase | ControlXC_Transchalcone | -2.3284 |
| TERG_04530 | hypothetical protein TERG_04530 | ElastinXE_Transchalcone | -1.7978 |
| TERG_04532 | hypothetical protein H100_04776 | ControlXC_Transchalcone | -2.5605 |
| TERG_04547 | aquaporin | ControlXKeratin | 5.1443 |
| TERG_04547 | aquaporin | KeratinXK_Transchalcone | -3.3562 |
| TERG_04558 | nucleoside diphosphate kinase | ControlXC_Transchalcone | 2.6563 |
| TERG_04558 | nucleoside diphosphate kinase | ControlXKeratin | 3.0630 |
| TERG_04574 | s-adenosylmethionine decarboxylase proenzyme | ElastinXE_Transchalcone | -1.0298 |
| TERG_04580 | nadp-specific glutamate dehydrogenase | ControlXKeratin | -2.5790 |
| TERG_04582 | extracellular cellulase allergen asp f7- | ControlXKeratin | 2.4761 |
| TERG_04592 | methionine type ii | ElastinXE_Transchalcone | 1.0596 |
| TERG_04594 | outer mitochondrial membrane protein porin | ControlXKeratin | 2.3285 |
| TERG_04598 | peroxisomal-coenzyme a synthetase | ControlXC_Transchalcone | 3.0313 |
| TERG_04610 | hypothetical protein TERG_04610 | ControlXKeratin | 2.7498 |
| TERG_04610 | hypothetical protein TERG_04610 | KeratinXK_Transchalcone | -3.3968 |
| TERG_04615 | hypothetical protein H105_04866 | ControlXElastin | 3.2504 |
| TERG_04615 | hypothetical protein H105_04866 | ControlXKeratin | 2.9549 |
| TERG_04616 | conserved hypothetical protein | ControlXElastin | 4.8172 |
| TERG_04616 | conserved hypothetical protein | ControlXKeratin | 5.4714 |
| TERG_04616 | conserved hypothetical protein | ElastinXE_Transchalcone | 1.0582 |
| TERG_04616 | conserved hypothetical protein | KeratinXK_Transchalcone | -2.3295 |
| TERG_04666 | f1f0-atp synthase regulatory factor stf2 | ControlXKeratin | 2.3570 |
| TERG_04666 | f1f0-atp synthase regulatory factor stf2 | ElastinXE_Transchalcone | -1.4718 |
| TERG_04676 | cell division control protein 10 | ControlXKeratin | 3.4776 |
| TERG_04682 | 3-hydroxyphenylacetate 6 hydroxylase | ElastinXE_Transchalcone | 1.2842 |
| TERG_04714 | mitochondrial phosphate carrier protein 2 | ElastinXE_Transchalcone | -1.3942 |
| TERG_04765 | mfs transporter | ControlXKeratin | -2.4484 |
| TERG_04766 | calcium proton exchanger | ControlXKeratin | 2.3311 |
| TERG_04769 | extracelular serine carboxypeptidase | ControlXKeratin | 2.5417 |
| TERG_04774 | isoflavone reductase family protein | ElastinXE_Transchalcone | 1.1490 |
| TERG_04775 | chitobiase | ControlXKeratin | 2.5302 |
| TERG_04778 | ribosome assembly and transport protein | ElastinXE_Transchalcone | -1.0919 |
| TERG_04809 | extracellular metalloproteinase 2 | ControlXKeratin | -3.0209 |
| TERG_04824 | hypothetical protein TERG_04824 | ControlXKeratin | 2.6208 |
| TERG_04851 | acyl binding protein family | ControlXKeratin | 2.9640 |
| TERG_04851 | acyl binding protein family | ElastinXE_Transchalcone | -1.2043 |
| TERG_04857 | ribose 5-phosphate isomerase | ControlXKeratin | -2.8522 |
| TERG_04862 | c6 sexual development transcription factor | ControlXElastin | 2.4185 |
| TERG_04862 | c6 sexual development transcription factor | ControlXKeratin | 3.3888 |
| TERG_04862 | c6 sexual development transcription factor | ElastinXE_Transchalcone | 1.1017 |
| TERG_04862 | c6 sexual development transcription factor | KeratinXK_Transchalcone | -3.5214 |
| TERG_04867 | sam and ph domain-containing protein | ControlXKeratin | 3.3857 |
| TERG_04867 | sam and ph domain-containing protein | KeratinXK_Transchalcone | -3.4831 |
| TERG_04883 | hypothetical protein TERG_04883 | ControlXKeratin | 3.2264 |
| TERG_04905 | rhodanese domain protein | ControlXKeratin | 2.7935 |
| TERG_04914 | spo7-like protein | ControlXKeratin | 2.4333 |
| TERG_04918 | chitin synthase | ControlXKeratin | -3.5097 |
| TERG_04938 | hypothetical protein TERG_04938 | ControlXKeratin | -2.6463 |
| TERG_04939 | hypothetical protein ARB_03846 | ControlXKeratin | -3.3824 |
| TERG_04966 | hypothetical protein TERG_04966 | ControlXKeratin | 2.3438 |
| TERG_04991 | nadh-ubiquinone oxidoreductase 64 kda subunit | ControlXKeratin | 2.5319 |
| TERG_04991 | nadh-ubiquinone oxidoreductase 64 kda subunit | KeratinXK_Transchalcone | -3.0267 |
| TERG_05035 | pentatricopeptide repeat protein | KeratinXK_Transchalcone | -2.6076 |
| TERG_05049 | nucleoside diphosphatase | ControlXKeratin | 2.4071 |
| TERG_05118 | actin cytoskeleton organization protein | ControlXKeratin | -2.3811 |
| TERG_05151 | domon-like type 9 carbohydrate-binding module domain-containing protein | ControlXElastin | 2.3976 |
| TERG_05151 | domon-like type 9 carbohydrate-binding module domain-containing protein | ElastinXE_Transchalcone | 1.0496 |
| TERG_05153 | mfs transporter | ControlXKeratin | -2.5031 |
| TERG_05172 | alternative nadh-dehydrogenase | ControlXC_Transchalcone | 2.9837 |
| TERG_05172 | alternative nadh-dehydrogenase | ElastinXE_Transchalcone | -1.7805 |
| TERG_05199 | mfs gliotoxin efflux transporter | ControlXKeratin | -2.4741 |
| TERG_05201 | integral membrane | KeratinXK_Transchalcone | 2.5381 |
| TERG_05229 | homeobox transcription | KeratinXK_Transchalcone | -2.4957 |
| TERG_05239 | dna polymerase lambda | KeratinXK_Transchalcone | 2.4898 |
| TERG_05245 | dna repair protein | ControlXKeratin | -2.6860 |
| TERG_05251 | phosphoglycerate kinase | ElastinXE_Transchalcone | -1.2230 |
| TERG_05274 | hypothetical protein H102_03943 | ControlXKeratin | 2.9859 |
| TERG_05294 | hypothetical protein TERG_05294 | ElastinXE_Transchalcone | -1.1012 |
| TERG_05310 | kynurenine 3-monooxygenase | ControlXKeratin | 3.1403 |
| TERG_05317 | lipase 1 | ControlXKeratin | 3.5282 |
| TERG_05360 | 50s ribosomal subunit l30 | ElastinXE_Transchalcone | -1.4450 |
| TERG_05429 | mfs transporter | ControlXKeratin | -3.4633 |
| TERG_05429 | mfs transporter | KeratinXK_Transchalcone | 2.6522 |
| TERG_05435 | might be a transmembrane protein | ControlXKeratin | -3.1574 |
| TERG_05435 | might be a transmembrane protein | KeratinXK_Transchalcone | 2.4115 |
| TERG_05484 | acyl- dehydrogenase | KeratinXK_Transchalcone | 2.6670 |
| TERG_05492 | hypothetical protein TERG_05492 | ControlXKeratin | 4.0682 |
| TERG_05518 | short chain dehydrogenase | ElastinXE_Transchalcone | 1.1995 |
| TERG_05540 | cytochrome p450 | ControlXC_Transchalcone | -2.4550 |
| TERG_05558 | ornithine decarboxylase | ElastinXE_Transchalcone | -1.3115 |
| TERG_05575 | mfs multidrug transporter | KeratinXK_Transchalcone | 3.0578 |
| TERG_05576 | cell wall glucanase | ElastinXE_Transchalcone | -1.1683 |
| TERG_05616 | hypothetical protein H113_03004 | KeratinXK_Transchalcone | -2.4190 |
| TERG_05617 | hypothetical protein H100_02996 | ControlXC_Transchalcone | -3.6309 |
| TERG_05617 | hypothetical protein H100_02996 | KeratinXK_Transchalcone | -2.4048 |
| TERG_05625 | glycoside hydrolase family 18 protein | ControlXC_Transchalcone | -3.0295 |
| TERG_05627 | domain-containing protein | ControlXC_Transchalcone | -3.1344 |
| TERG_05627 | domain-containing protein | ControlXKeratin | 4.2253 |
| TERG_05652 | leucine aminopeptidase 1 | ControlXElastin | 3.0096 |
| TERG_05652 | leucine aminopeptidase 1 | ControlXKeratin | 4.3723 |
| TERG_05659 | double-stranded rna-binding-like | ControlXKeratin | 3.5993 |
| TERG_05659 | double-stranded rna-binding-like | KeratinXK_Transchalcone | -3.1552 |
| TERG_05700 | hypothetical protein H100_03088 | ControlXElastin | 2.5176 |
| TERG_05701 | c4-dicarboxylate transporter malic acid transport | ControlXKeratin | -2.7850 |
| TERG_05735 | dipeptidyl peptidase 4 | ControlXKeratin | 3.0944 |
| TERG_05741 | duf1716 domain-containing protein | ControlXElastin | 2.4062 |
| TERG_05744 | gtp-binding protein | ControlXKeratin | 3.0773 |
| TERG_05744 | gtp-binding protein | ElastinXE_Transchalcone | 1.1512 |
| TERG_05744 | gtp-binding protein | KeratinXK_Transchalcone | -2.7813 |
| TERG_05789 | nadh-cytochrome b5 reductase 2 | ControlXKeratin | 2.3587 |
| TERG_05793 | nuclear factor erythroid 2-related factor 2 | KeratinXK_Transchalcone | -2.5491 |
| TERG_05799 | chlorophyll synthesis pathway protein | ControlXElastin | 2.4669 |
| TERG_05804 | ef hand domain-containing protein | ElastinXE_Transchalcone | 1.3045 |
| TERG_05806 | methionine type i | ControlXElastin | 5.6431 |
| TERG_05806 | methionine type i | ControlXKeratin | 4.3310 |
| TERG_05808 | conserved hypothetical protein | ControlXC_Transchalcone | -2.5251 |
| TERG_05808 | conserved hypothetical protein | ControlXKeratin | 2.3985 |
| TERG_05808 | conserved hypothetical protein | KeratinXK_Transchalcone | -2.9100 |
| TERG_05823 | integral membrane protein | ElastinXE_Transchalcone | 1.2266 |
| TERG_05823 | integral membrane protein | KeratinXK_Transchalcone | -2.4790 |
| TERG_05837 | gpr fun34 family protein | ControlXC_Transchalcone | 2.4291 |
| TERG_05868 | opsin 1 | ControlXKeratin | -2.8082 |
| TERG_05885 | domain protein | ElastinXE_Transchalcone | -1.5021 |
| TERG_05923 | metallopeptidase | ControlXKeratin | -2.6087 |
| TERG_05955 | heat shock factor-binding protein 1 | ControlXKeratin | 2.3255 |
| TERG_05971 | ribonucleoside-diphosphate reductase small chain | ControlXC_Transchalcone | -3.5519 |
| TERG_05987 | gtp-binding protein | ControlXC_Transchalcone | 3.0220 |
| TERG_05987 | gtp-binding protein | ElastinXE_Transchalcone | -1.8980 |
| TERG_05988 | glycine-rich cell wall structural protein 1 | KeratinXK_Transchalcone | 3.3753 |
| TERG_06022 | hypothetical protein H100_02258 | ControlXElastin | 2.4245 |
| TERG_06022 | hypothetical protein H100_02258 | ControlXKeratin | 2.5211 |
| TERG_06059 | helicase swr1 | ControlXKeratin | 2.5932 |
| TERG_06070 | polysaccharide synthase cps1 | ControlXKeratin | 2.8196 |
| TERG_06116 | indoleamine -dioxygenase | ControlXC_Transchalcone | 2.4240 |
| TERG_06144 | cell wall serine-threonine-rich galactomannoprotein mp1 | ControlXKeratin | 2.4350 |
| TERG_06149 | nima interactive protein | ControlXC_Transchalcone | -4.3376 |
| TERG_06149 | nima interactive protein | ControlXKeratin | 2.4803 |
| TERG_06160 | nitrite copper-containing | ControlXKeratin | 2.4037 |
| TERG_06186 | protein disulfide-isomerase domain-containing protein | KeratinXK_Transchalcone | 2.4442 |
| TERG_06222 | myb dna-binding domain-containing protein | ControlXC_Transchalcone | 2.3411 |
| TERG_06228 | hypothetical protein TERG_06228 | ControlXKeratin | 2.3731 |
| TERG_06239 | hypothetical protein TERG_06239 | KeratinXK_Transchalcone | 2.6427 |
| TERG_06242 | glucan -beta-glucosidase | ControlXElastin | 3.5209 |
| TERG_06242 | glucan -beta-glucosidase | ControlXKeratin | 2.9318 |
| TERG_06242 | glucan -beta-glucosidase | ElastinXE_Transchalcone | 1.1970 |
| TERG_06243 | glutathione s-transferase | ControlXElastin | 2.6205 |
| TERG_06243 | glutathione s-transferase | ControlXKeratin | 3.0060 |
| TERG_06267 | hypothetical protein TERG_06267 | ControlXKeratin | 2.4774 |
| TERG_06267 | hypothetical protein TERG_06267 | KeratinXK_Transchalcone | -2.7009 |
| TERG_06296 | hypothetical protein H100_02537 | ControlXKeratin | 2.7271 |
| TERG_06347 | protein | ControlXKeratin | 2.4473 |
| TERG_06355 | acetyltransferase | ControlXKeratin | -2.5621 |
| TERG_06375 | hypothetical protein H100_02625 | ControlXKeratin | -2.5129 |
| TERG_06437 | ribosomal protein l28e | ControlXKeratin | -3.0997 |
| TERG_06504 | conserved hypothetical protein | KeratinXK_Transchalcone | -3.2849 |
| TERG_06505 | hsp70-like protein | ControlXKeratin | 2.4555 |
| TERG_06505 | hsp70-like protein | ElastinXE_Transchalcone | -1.0988 |
| TERG_06552 | aspartic-type endopeptidase | ControlXKeratin | 2.5415 |
| TERG_06552 | aspartic-type endopeptidase | ElastinXE_Transchalcone | 1.0259 |
| TERG_06578 | elongation factor 1-gamma | KeratinXK_Transchalcone | 2.3876 |
| TERG_06585 | hypothetical protein ARB_04141 | ControlXKeratin | -4.0397 |
| TERG_06594 | duf1183 domain-containing protein | KeratinXK_Transchalcone | 2.9104 |
| TERG_06607 | serine-rich | KeratinXK_Transchalcone | -2.9773 |
| TERG_06637 | actin | ControlXC_Transchalcone | 2.3480 |
| TERG_06637 | actin | ElastinXE_Transchalcone | -1.4410 |
| TERG_06638 | class v chitinase | ControlXKeratin | 2.6254 |
| TERG_06646 | arrestin | ElastinXE_Transchalcone | -1.1051 |
| TERG_06701 | gamma-glutamyltransferase | ControlXC_Transchalcone | 2.7253 |
| TERG_06707 | arginase | ElastinXE_Transchalcone | -1.2775 |
| TERG_06741 | ubiquitin c-terminal hydrolase | ElastinXE_Transchalcone | -1.4024 |
| TERG_06744 | fructose-bisphosphate aldolase 1 | ControlXKeratin | 2.3484 |
| TERG_06754 | cytochrome c mitochondrial | ControlXKeratin | 2.3413 |
| TERG_06756 | domain protein | ControlXC_Transchalcone | -2.5131 |
| TERG_06792 | hypothetical protein TERG_06792 | ElastinXE_Transchalcone | 1.0193 |
| TERG_06870 | heat shock protein sti1 | ElastinXE_Transchalcone | -1.0912 |
| TERG_06890 | duf636 domain protein | ControlXKeratin | -2.6909 |
| TERG_06906 | dna mismatch repair protein msh6 | ControlXElastin | -4.1137 |
| TERG_06906 | dna mismatch repair protein msh6 | ControlXKeratin | -4.1650 |
| TERG_06962 | heat shock protein | ElastinXE_Transchalcone | -1.1203 |
| TERG_06963 | hsp90-like protein | ControlXKeratin | 2.3955 |
| TERG_06963 | hsp90-like protein | ElastinXE_Transchalcone | -1.0536 |
| TERG_06986 | exo-beta- -glucanase exg0 | ControlXElastin | 3.7872 |
| TERG_06986 | exo-beta- -glucanase exg0 | ControlXKeratin | 2.7271 |
| TERG_07002 | 60s ribosomal protein l17 | ControlXC_Transchalcone | 3.1831 |
| TERG_07006 | pab-dependent poly -specific ribonuclease subunit pan3 | ControlXKeratin | 2.9259 |
| TERG_07035 | glutathione synthetase atp-binding domain-like protein | ControlXC_Transchalcone | -2.5809 |
| TERG_07053 | hypothetical protein H100_01401 | ControlXKeratin | -2.4992 |
| TERG_07053 | hypothetical protein H100_01401 | ElastinXE_Transchalcone | -1.0470 |
| TERG_07054 | autophagy protein | KeratinXK_Transchalcone | -2.4070 |
| TERG_07083 | hypothetical protein H105_01451 | ElastinXE_Transchalcone | -1.1152 |
| TERG_07089 | gpi-anchored wall transfer protein 1 | ControlXKeratin | 2.6767 |
| TERG_07097 | camk camkl kin4 protein kinase | KeratinXK_Transchalcone | -2.5538 |
| TERG_07127 | homocitrate mitochondrial | ControlXC_Transchalcone | 2.6918 |
| TERG_07136 | farnesyltransferase beta subunit ram1 | ControlXC_Transchalcone | -2.4260 |
| TERG_07136 | farnesyltransferase beta subunit ram1 | KeratinXK_Transchalcone | -3.2165 |
| TERG_07143 | potassium sodium efflux p-type fungal-type | ControlXC_Transchalcone | 2.4493 |
| TERG_07143 | potassium sodium efflux p-type fungal-type | KeratinXK_Transchalcone | 2.3643 |
| TERG_07159 | prenyltransferase alpha subunit | ControlXKeratin | 2.5343 |
| TERG_07193 | kynurenine aminotransferase | ControlXKeratin | 3.0900 |
| TERG_07200 | c2 domain-containing protein | KeratinXK_Transchalcone | 2.5396 |
| TERG_07205 | nadh-ubiquinone oxidoreductase 51 kda mitochondrial | ElastinXE_Transchalcone | -1.4027 |
| TERG_07234 | hypothetical protein H100_01597 | ElastinXE_Transchalcone | 1.1388 |
| TERG_07267 | hypothetical protein TERG_07267 | ElastinXE_Transchalcone | 1.0910 |
| TERG_07283 | gaba permease | KeratinXK_Transchalcone | -2.4800 |
| TERG_07363 | hypothetical protein TERG_07363 | KeratinXK_Transchalcone | 2.6689 |
| TERG_07379 | leucine rich repeat domain protein | ControlXKeratin | 2.9896 |
| TERG_07379 | leucine rich repeat domain protein | ElastinXE_Transchalcone | 1.4063 |
| TERG_07396 | dna-directed rna polymerase ii largest subunit | ElastinXE_Transchalcone | 1.2429 |
| TERG_07406 | a chain structure of citrinum alpha -mannosidase reveals the basis for differences in specificity of the er and golgi class i enzymes | ControlXElastin | 4.0231 |
| TERG_07427 | homeobox and c2h2 transcription factor | ControlXElastin | 3.0156 |
| TERG_07427 | homeobox and c2h2 transcription factor | ControlXKeratin | 2.8806 |
| TERG_07456 | cell wall protein | ElastinXE_Transchalcone | 1.0635 |
| TERG_07477 | copper-sulfate regulated protein 1 | ControlXC_Transchalcone | -2.4739 |
| TERG_07477 | copper-sulfate regulated protein 1 | ElastinXE_Transchalcone | -1.0353 |
| TERG_07478 | transcription factor TFIIIB complex subunit brf1 | KeratinXK_Transchalcone | -2.9004 |
| TERG_07504 | carbohydrate-binding protein | ControlXC_Transchalcone | -2.8457 |
| TERG_07504 | carbohydrate-binding protein | ControlXElastin | -2.7089 |
| TERG_07504 | carbohydrate-binding protein | KeratinXK_Transchalcone | -4.2470 |
| TERG_07518 | histidyl-trna synthetase | ControlXC_Transchalcone | 2.5009 |
| TERG_07543 | hypothetical protein H100_01125 | ControlXKeratin | 2.8237 |
| TERG_07543 | hypothetical protein H100_01125 | KeratinXK_Transchalcone | -2.3687 |
| TERG_07556 | hypothetical protein TERG_07556 | ControlXKeratin | -2.4588 |
| TERG_07563 | polynucleotide kinase 3 -phosphatase | ControlXKeratin | -2.4242 |
| TERG_07565 | hypothetical protein TERG_07565 | ControlXKeratin | -2.5150 |
| TERG_07570 | g-protein signaling | ElastinXE_Transchalcone | -1.2327 |
| TERG_07625 | amino acid transporter | ControlXKeratin | 2.5875 |
| TERG_07626 | hypothetical protein TERG_07626 | ElastinXE_Transchalcone | -1.7014 |
| TERG_07636 | dual specificity phosphatase | ElastinXE_Transchalcone | -1.6712 |
| TERG_07658 | hsp88-like protein | ElastinXE_Transchalcone | -1.0420 |
| TERG_07665 | hypothetical protein H100_01245 | ControlXKeratin | -2.3675 |
| TERG_07699 | ring finger domain protein | ControlXKeratin | -2.6405 |
| TERG_07705 | hypothetical protein H113_01289 | ControlXKeratin | 2.6447 |
| TERG_07705 | hypothetical protein H113_01289 | ElastinXE_Transchalcone | 1.0255 |
| TERG_07716 | hypothetical protein H100_01297 | ControlXKeratin | 2.6387 |
| TERG_07720 | hypothetical protein TERG_07720 | KeratinXK_Transchalcone | -2.7698 |
| TERG_07754 | hypothetical protein H100_00631 | ControlXKeratin | 3.2461 |
| TERG_07754 | hypothetical protein H100_00631 | KeratinXK_Transchalcone | -3.4199 |
| TERG_07783 | oligopeptide transporter | ControlXKeratin | 2.6522 |
| TERG_07796 | short-chain dehydrogenase reductase family | ControlXElastin | 2.4663 |
| TERG_07796 | short-chain dehydrogenase reductase family | KeratinXK_Transchalcone | 2.7793 |
| TERG_07802 | siderophore biosynthesis acetylase | ElastinXE_Transchalcone | -1.0089 |
| TERG_07810 | hypothetical protein TERG_07810 | KeratinXK_Transchalcone | -2.3948 |
| TERG_07832 | protein | ElastinXE_Transchalcone | -1.1843 |
| TERG_07834 | alpha-actinin | ControlXElastin | 2.9152 |
| TERG_07834 | alpha-actinin | ControlXKeratin | 2.5515 |
| TERG_07842 | hypothetical protein TRV_04011 | ControlXKeratin | 2.7710 |
| TERG_07842 | hypothetical protein TRV_04011 | ElastinXE_Transchalcone | 1.5360 |
| TERG_07849 | amp-binding enzyme | ElastinXE_Transchalcone | -1.1274 |
| TERG_07859 | ubiquitin c-terminal hydrolase l3 | ControlXKeratin | -2.5460 |
| TERG_07870 | alpha- -mannosyltransferase subunit | ControlXKeratin | -2.3988 |
| TERG_07875 | integral membrane protein | ControlXKeratin | 3.2618 |
| TERG_07875 | integral membrane protein | ElastinXE_Transchalcone | 1.2043 |
| TERG_07904 | tubulin beta chain | KeratinXK_Transchalcone | -2.9634 |
| TERG_07905 | 1-pyrroline-5-carboxylate dehydrogenase | ControlXKeratin | 3.4758 |
| TERG_07919 | thioesterase family protein | ControlXC_Transchalcone | -2.4016 |
| TERG_07919 | thioesterase family protein | ControlXKeratin | -2.7080 |
| TERG_07934 | integral membrane | ControlXC_Transchalcone | -2.4769 |
| TERG_07934 | integral membrane | KeratinXK_Transchalcone | -2.7479 |
| TERG_07944 | 60s ribosomal protein l6 | KeratinXK_Transchalcone | -2.5170 |
| TERG_07948 | dna mismatch repair protein msh5 | ElastinXE_Transchalcone | -1.3045 |
| TERG_07963 | hypothetical protein TESG_05670 | KeratinXK_Transchalcone | -2.4764 |
| TERG_07978 | hypothetical protein TERG_07978 | ControlXC_Transchalcone | 2.3420 |
| TERG_07982 | hypothetical protein TRV_01878 | ControlXKeratin | -2.8193 |
| TERG_07997 | acetamidase | ControlXKeratin | -3.0931 |
| TERG_08004 | cytochrome p450 | KeratinXK_Transchalcone | 2.6412 |
| TERG_08029 | hmg box protein | ControlXElastin | 2.9456 |
| TERG_08047 | hypothetical protein TERG_08047 | ControlXKeratin | 2.6343 |
| TERG_08076 | integral membrane protein | KeratinXK_Transchalcone | 2.4380 |
| TERG_08116 | von willebrand factor | ElastinXE_Transchalcone | 1.1148 |
| TERG_08138 | trna processing endoribonuclease | ControlXKeratin | -2.7382 |
| TERG_08139 | nad dependent epimerase dehydratase family protein | ElastinXE_Transchalcone | -1.0764 |
| TERG_08140 | 2-heptaprenyl- -naphthoquinone | ElastinXE_Transchalcone | -1.0556 |
| TERG_08161 | hypothetical protein TERG_08161 | ControlXC_Transchalcone | 2.6747 |
| TERG_08161 | hypothetical protein TERG_08161 | ElastinXE_Transchalcone | -1.2671 |
| TERG_08180 | oxalate decarboxylase | ElastinXE_Transchalcone | 1.7187 |
| TERG_08201 | subtilisin-like protease 5 | ControlXC_Transchalcone | -2.8951 |
| TERG_08201 | subtilisin-like protease 5 | ControlXKeratin | 3.1787 |
| TERG_08235 | long chain fatty alcohol oxidase | ControlXC_Transchalcone | -2.4640 |
| TERG_08247 | hypothetical protein TERG_08247 | ElastinXE_Transchalcone | -1.0739 |
| TERG_08261 | glutamate decarboxylase | KeratinXK_Transchalcone | -2.9500 |
| TERG_08264 | l-xylulose reductase | KeratinXK_Transchalcone | -2.5599 |
| TERG_08286 | proline-rich- partial | ControlXKeratin | 2.4933 |
| TERG_08352 | sodium calcium transporter | ControlXKeratin | -2.4449 |
| TERG_08353 | cytochrome p450 | ControlXElastin | -3.3457 |
| TERG_08353 | cytochrome p450 | ControlXKeratin | -3.7813 |
| TERG_08353 | cytochrome p450 | ElastinXE_Transchalcone | -1.4468 |
| TERG_08378 | chromatin modification-like protein eaf6 | ControlXElastin | 2.7743 |
| TERG_08396 | hypothetical protein H100_00257 | KeratinXK_Transchalcone | -3.7734 |
| TERG_08405 | leucine aminopeptidase 2 | ControlXKeratin | 3.0553 |
| TERG_08436 | hypothetical protein H100_00297 | ElastinXE_Transchalcone | -1.4871 |
| TERG_08451 | upf0052 domain-containing protein | KeratinXK_Transchalcone | 2.3303 |
| TERG_08468 | hydroxyacylglutathione hydrolase | ControlXKeratin | -3.7920 |
| TERG_08499 | mitochondrial distribution and morphology protein 10 | ControlXC_Transchalcone | -2.4456 |
| TERG_08508 | hypothetical protein TERG_08508 | ControlXKeratin | -2.4733 |
| TERG_08511 | hypothetical protein TERG_08511 | ControlXC_Transchalcone | -3.2599 |
| TERG_08511 | hypothetical protein TERG_08511 | ControlXKeratin | -2.5900 |
| TERG_08539 | lipase serine esterase | ControlXKeratin | -2.3226 |
| TERG_08545 | c-4 methyl sterol oxidase | ElastinXE_Transchalcone | -1.4734 |
| TERG_08553 | ran exchange factor prp20 pim1 | ControlXElastin | -2.6717 |
| TERG_08553 | ran exchange factor prp20 pim1 | ControlXKeratin | -2.5473 |
| TERG_08557 | carboxypeptidase s1 | ControlXKeratin | 2.8559 |
| TERG_08562 | hypothetical protein TERG_08562 | ControlXKeratin | -2.4462 |
| TERG_08563 | hypothetical protein TERG_08563 | ControlXKeratin | -2.7571 |
| TERG_08613 | ABC multidrug transporter | KeratinXK_Transchalcone | 2.4969 |
| TERG_08614 | hypothetical protein TERG_08614 | KeratinXK_Transchalcone | -3.1078 |
| TERG_08619 | siderophore iron transporter mirb | KeratinXK_Transchalcone | -2.3749 |
| TERG_08666 | c2h2 type zinc finger containing protein | ControlXC_Transchalcone | 2.4338 |
| TERG_08666 | c2h2 type zinc finger containing protein | ControlXKeratin | 4.3947 |
| TERG_08666 | c2h2 type zinc finger containing protein | KeratinXK_Transchalcone | -2.5243 |
| TERG_08708 | wd domain-containing protein | ControlXC_Transchalcone | -2.4454 |
| TERG_08708 | wd domain-containing protein | KeratinXK_Transchalcone | -2.7806 |
| TERG_08709 | protein | ControlXC_Transchalcone | -2.4784 |
| TERG_08709 | protein | KeratinXK_Transchalcone | -2.8772 |
| TERG_08761 | lipase esterase family protein | KeratinXK_Transchalcone | 2.3457 |
| TERG_08787 | aminotransferase family protein | ControlXC_Transchalcone | 2.6599 |
| TERG_08787 | aminotransferase family protein | ControlXElastin | -2.3348 |
| TERG_08794 | pyruvate dehydrogenase kinase | KeratinXK_Transchalcone | -2.4467 |
| TERG_08801 | hypothetical protein TERG_08801 | ElastinXE_Transchalcone | 1.0007 |
| TERG_08854 | mitochondrial hypoxia responsive domain-containing protein | ControlXKeratin | -2.5188 |
| TERG_08855 | hypothetical protein TERG_08855 | ControlXKeratin | -2.5202 |
| TERG_08869 | hypothetical protein H103_03034 | KeratinXK_Transchalcone | -2.5473 |
| TERG_08887 | adenine phosphoribosyltransferase | ElastinXE_Transchalcone | -1.1434 |
| TERG_08941 | methyltransferase | ControlXKeratin | 2.7592 |
| TERG_08968 | rna export mediator gle1 | ControlXC_Transchalcone | -2.9938 |
| TERG_08969 | cu-zn superoxide dismutase | ElastinXE_Transchalcone | -2.0157 |
| TERG_11518 | glucokinase | ControlXElastin | 2.3447 |
| TERG_11538 | 3-oxoacyl-(acyl-carrier-protein) reductase | ControlXC_Transchalcone | -3.4080 |
| TERG_11538 | 3-oxoacyl-(acyl-carrier-protein) reductase | KeratinXK_Transchalcone | -2.8976 |
| TERG_11539 | 3-oxoacyl-(acyl-carrier-protein) reductase | ControlXC_Transchalcone | -2.4776 |
| TERG_11563 | beta-lactamase family protein | ControlXKeratin | -2.5158 |
| TERG_11621 | hypothetical protein H100_08206 | KeratinXK_Transchalcone | 2.3951 |
| TERG_11628 | glutamine synthetase | ControlXKeratin | -2.4536 |
| TERG_11673 | ubiquitin carboxyl-terminal hydrolase | ControlXKeratin | -2.6522 |
| TERG_11710 | hypothetical protein H100_08860 | KeratinXK_Transchalcone | 2.5280 |
| TERG_11745 | integral membrane protein | ControlXC_Transchalcone | -4.7386 |
| TERG_11745 | integral membrane protein | ControlXKeratin | -3.7656 |
| TERG_11746 | integral membrane protein | ControlXC_Transchalcone | -3.8281 |
| TERG_11746 | integral membrane protein | ControlXKeratin | -3.2146 |
| TERG_11746 | integral membrane protein | ElastinXE_Transchalcone | 1.0519 |
| TERG_11747 | hypothetical protein | ControlXKeratin | -2.7116 |
| TERG_11747 | hypothetical protein | KeratinXK_Transchalcone | 5.1822 |
| TERG_11753 | mfs transporter | ControlXElastin | 5.1126 |
| TERG_11753 | mfs transporter | ControlXKeratin | 4.6062 |
| TERG_11813 | fad binding domain-containing protein | KeratinXK_Transchalcone | 2.5721 |
| TERG_11814 | fad dependent | KeratinXK_Transchalcone | 3.4108 |
| TERG_11852 | glyoxalase family protein | KeratinXK_Transchalcone | -2.4466 |
| TERG_11886 | copper radical oxidase | ControlXKeratin | 4.0504 |
| TERG_11895 | polyketide synthase | ControlXC_Transchalcone | -2.4850 |
| TERG_11944 | hypothetical protein H113_05373 | ControlXC_Transchalcone | -2.8448 |
| TERG_11944 | hypothetical protein H113_05373 | KeratinXK_Transchalcone | -3.3198 |
| TERG_11963 | dead deah box rna helicase | ControlXC_Transchalcone | -2.9032 |
| TERG_11997 | potassium transporter | ControlXKeratin | 2.6318 |
| TERG_12029 | hypothetical protein H100_05938 | KeratinXK_Transchalcone | 2.3321 |
| TERG_12038 | protein phosphatase regulatory subunit | ControlXKeratin | -3.0994 |
| TERG_12107 | 1,4-alpha-glucan-branching enzyme | ControlXC_Transchalcone | -2.5216 |
| TERG_12156 | hypothetical protein H100_04777 | ControlXKeratin | -3.6304 |
| TERG_12156 | hypothetical protein H100_04777 | ElastinXE_Transchalcone | -1.1097 |
| TERG_12156 | hypothetical protein H100_04777 | KeratinXK_Transchalcone | 2.8041 |
| TERG_12169 | nuclear pore complex subunit | ElastinXE_Transchalcone | -1.0974 |
| TERG_12204 | rna polymerase i specific transcription initiation factor | KeratinXK_Transchalcone | 2.3840 |
| TERG_12213 | hypothetical protein H100_03617 | ElastinXE_Transchalcone | -1.2148 |
| TERG_12228 | hypothetical protein H100_03711 | ControlXKeratin | 2.5458 |
| TERG_12269 | dna-binding 42 kda | KeratinXK_Transchalcone | -2.3534 |
| TERG_12287 | fungal specific transcription factor domain protein | ElastinXE_Transchalcone | -1.0154 |
| TERG_12291 | mitochondrial cytochrome | ElastinXE_Transchalcone | -1.2116 |
| TERG_12292 | hypothetical protein H100_02865 | ControlXKeratin | 2.8659 |
| TERG_12320 | hypothetical protein H100_03043 | ControlXKeratin | 2.5516 |
| TERG_12351 | hypothetical protein H100_03320 | ControlXKeratin | 2.4861 |
| TERG_12351 | hypothetical protein H100_03320 | ElastinXE_Transchalcone | -1.0316 |
| TERG_12351 | hypothetical protein H100_03320 | KeratinXK_Transchalcone | 2.4977 |
| TERG_12434 | hypothetical protein H100_01872 | ControlXElastin | 3.5288 |
| TERG_12470 | ribonucleoprotein | ControlXC_Transchalcone | -2.3674 |
| TERG_12491 | lipase | ElastinXE_Transchalcone | -1.3201 |
| TERG_12501 | hypothetical protein H113_01357 | KeratinXK_Transchalcone | -2.5122 |
| TERG_12525 | fatty acid oxygenase | ControlXElastin | 2.3961 |
| TERG_12565 | hypothetical protein H100_01156 | KeratinXK_Transchalcone | -2.5467 |
| TERG_12567 | hypothetical protein H113_01189 | ElastinXE_Transchalcone | 1.0858 |
| TERG_12603 | hypothetical protein H100_00717 | KeratinXK_Transchalcone | -3.5935 |
| TERG_12606 | secreted dipeptidyl peptidase | ControlXKeratin | 2.3298 |
| TERG_12615 | ankyrin repeat domain-containing protein 28 | KeratinXK_Transchalcone | -2.5493 |
| TERG_12687 | ubiquitin ligase subunit | KeratinXK_Transchalcone | 2.6958 |
